# Transforming tumor microenvironments: nanotechnology and gene therapy in cellular signaling and epigenetic insight into chemo-resistance

**DOI:** 10.1186/s13046-026-03720-8

**Published:** 2026-05-04

**Authors:** Prashant Sharma, Nguyen Phuong Thuy, Israrul H. Ansari, Ravi Mani Tripathi, Mrinalini Kala, Mostafa H. Elberry, Neelesh Sharma, Sung-Jin Lee

**Affiliations:** 1 Department of Child Health, University of Arizona College of Medicine-Phoenix, ABC1 Building, 425 N 5Th Street, Phoenix, AZ 85004 USA; 2 Faculty of Medical Technology, Hanoi Medical University, Building A7, No.1 Ton That Tung, Hanoi, 100000 Vietnam; 3 Department of Pediatrics, Division of Pediatric Hematology, Oncology and Bone Marrow Transplant, 4136 Wisconsin Institutes for Medical Research I, University of Wisconsin, Madison, WI 53706 USA; 4 Amity Institute of Nanotechnology, Amity University Uttar Pradesh, Sector 125, Noida, UP 201303 India; 5 Department Internal Medicine, University of Arizona College of Medicine-Phoenix, 475 N. 5th Street, Phoenix, AZ 85004 USA; 6 Division of Veterinary Medicine, Faculty of Veterinary Sciences and Animal Husbandry, Sher- E-Kashmir University of Agricultural Sciences and Technology of Jammu, Ranbir Singh Pura, Union Territory of Jammu and Kashmir 181 102 India; 7 Department of Applied Animal Science, College of Animal Life Sciences, Kangwon National University, Chuncheon, Republic of Korea

**Keywords:** Chemoresistance, Nanotechnology, Gene Therapy, Epigenetic, Tumor Microenvironment, Regulatory Challenges, FDA, CRISPR

## Abstract

Chemoresistance remains the primary cause of cancer treatment failure, yet current understanding remains fragmented across isolated mechanistic studies. This review provides a unified framework linking tumor microenvironment (TME) signaling, epigenetic reprogramming, and nanotherapeutic intervention as an integrated axis driving and potentially reversing chemoresistance. We systematically examine how TME components: hypoxia (HIF-1α pathway), acidosis, cancer-associated fibroblasts (TGF-β/PDGF signaling), and immune cells (NF-κB-mediated immunosuppression) activate signaling cascades that directly interface with epigenetic machinery. These TME-activated pathways recruit DNA methyltransferases, histone-modifying enzymes, and regulate microRNA (miRNA) networks, establishing stable resistant phenotypes including epithelial-mesenchymal transition, cancer stem cells, and metabolic adaptation. Critically, miRNA dysregulation serves as a central integrator, creating bidirectional crosstalk between signaling pathways and epigenetic modifications through self-reinforcing circuits. Unlike previous reviews focusing on isolated resistance mechanisms, we demonstrate how this integrated TME-epigenetic axis creates specific therapeutic vulnerabilities exploitable through rationally designed nanotechnology platforms delivering epigenetic modulators (DNMT inhibitors, HDAC inhibitors, EZH2 inhibitors) and gene therapy tools (CRISPR-Cas9 epigenetic editors, miRNA mimics/antagomirs). We critically evaluate clinical translation challenges, including EPR effect heterogeneity, delivery barriers, and biomarker gaps, providing a balanced perspective on both potential and obstacles. This mechanistic framework guides the development of next-generation combination therapies targeting multiple nodes within the TME-epigenetic-nanotherapy axis.

**Graphical Abstract:**

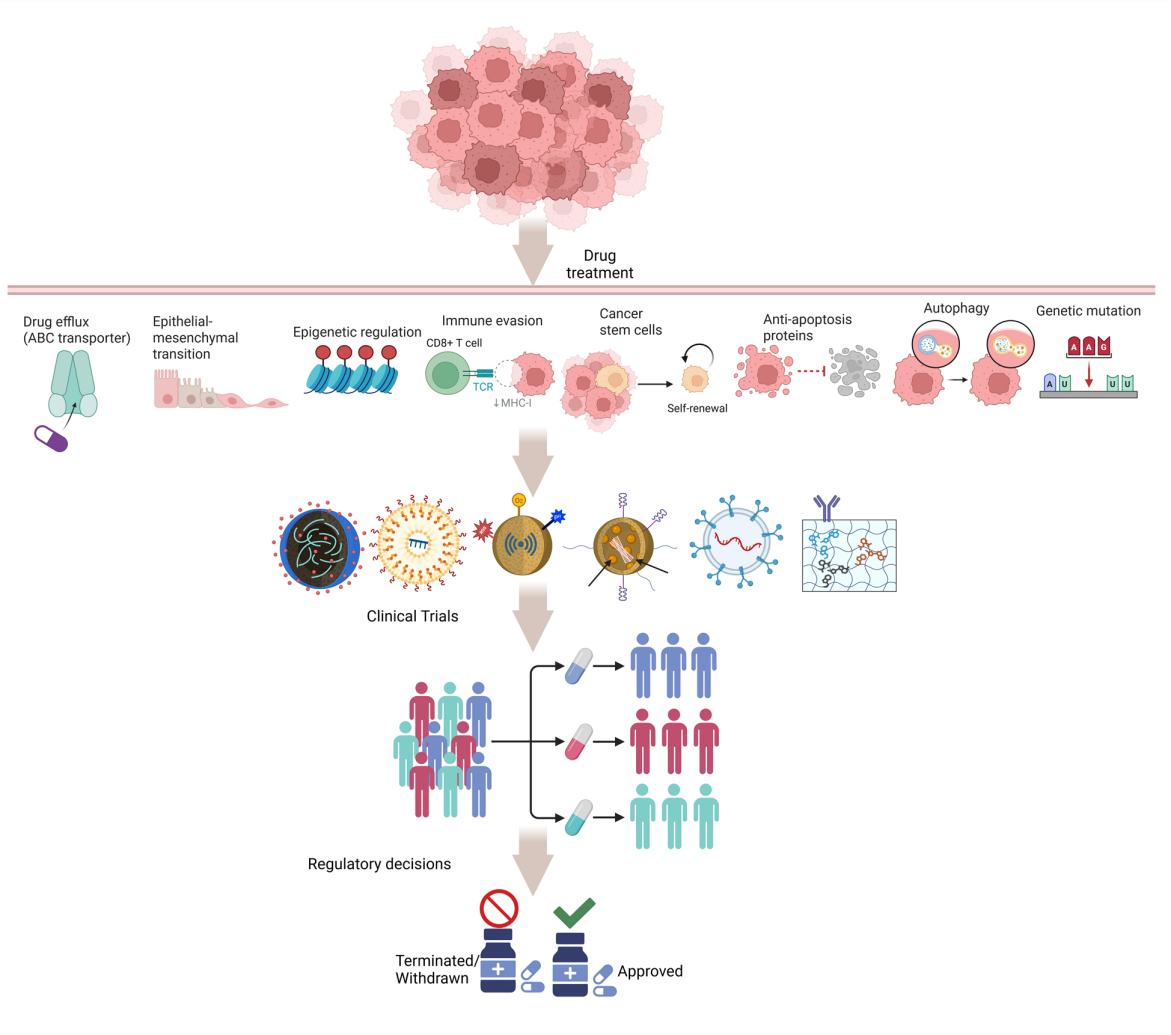

## Highlights

Explores how tumor microenvironment signals drive cancer chemoresistance.

Reviews nanotechnology and gene therapy to overcome drug resistance.

Details the role of epigenetic reprogramming in therapeutic failure.

Summarizes clinical trials specifically targeting chemoresistance

## Introduction

Chemoresistance, the failure of cancer cells to respond to chemotherapeutic drugs, is a major cause of cancer treatment failure, accounting for up to 80–90% of cancer-related mortality in advanced disease [1, 2]. This phenomenon may be present from the outset (intrinsic resistance) or develop under therapy (acquired resistance), and it underlies most cases of tumor relapse and poor survival outcomes [3]. Tackling chemoresistance is therefore pivotal for improving patient outcomes. Tumor cells evade drugs through multiple mechanisms: genetic mutation, activation of prosurvival signaling pathways, epigenetic reprogramming, metabolic adaptation, enhanced DNA repair, evasion of apoptosis, and involvement of the tumor microenvironment (TME) [1, 4, 5]. The TME, which is composed of hypoxic and acidic niches, stromal cells, fibroblasts and immune cells, the extracellular matrix, and soluble factors, is now recognized as equally important as intrinsic tumor factors in driving drug resistance [5–7]. Indeed, cancer cells depend on constant support from their microenvironment, and adaptive interactions between tumor cells and TME components promote survival under therapeutic stress [8, 9]. To overcome chemoresistance, modern research is exploring strategies that transcend conventional chemotherapy. Two promising and complementary approaches are emerging: nanotechnology-driven drug delivery systems and gene therapy interventions. Cellular signaling pathways that regulate proliferation, survival, and stress responses, such as the PI3K/AKT, MAPK/ERK, Wnt/β-catenin, Notch, and NF-κB pathways, often become dysregulated in drug-resistant cancers [10–14]. Moreover, epigenetic alterations such as DNA methylation, histone modifications, and noncoding RNAs can stably reprogram gene expression to favor survival, EMT, and stemness, further reinforcing resistance [1, 15–17]. Notably, there is extensive crosstalk between signaling and epigenetic networks, and oncogenic signaling pathways can induce epigenetic changes and vice versa, creating self-reinforcing loops that stabilize and perpetuate the resistant phenotype [1, 10, 16, 18]. The TME adds another layer of complexity by imposing hypoxia, nutrient deprivation and acidosis, immune suppression, and mechanical stress, all of which activate prosurvival signaling and epigenetic adaptive responses in cancer cells [19–22]. These insights point to the need for combination therapeutic strategies that target both the internal circuits of cancer cells and their microenvironmental context (Fig. 2).

Nanotechnology offers a great opportunity to transform the TME and improve drug delivery, leading to enhanced drug efficacy [23–25]. Novel nanocarriers have been formulated to bypass or exploit TME barriers, for example, releasing drugs in acidic or hypoxic zones or specifically targeting cancer stem cells and immunosuppressive cells in the TME [19, 26–29].

By enhancing the targeted localization and controlled release of therapeutic agents, nanocarriers have demonstrated the potential to overcome key physical and physiological barriers in preclinical models, including limited tumor penetration and the activity of drug efflux pumps, though clinical translation warrants further investigation. In parallel, gene therapy tools provide opportunities to directly modulate the molecular drivers of resistance. The development of genome editing tools such as CRISPR/Cas9 genome editing can knock out or correct genes conferring chemoresistance, including those encoding efflux transporters or mutant oncogenes [30–33]. Another approach involves the use of RNA interference RNAs (siRNAs), microRNAs (miRNAs), or antisense oligonucleotides to silence prosurvival genes transiently [1, 34–36]. The recent emergence of CRISPR-based epigenetic editors, such as deactivated Cas9 proteins fused to demethylase or deacetylase enzymes, has enabled locus-specific reprogramming of the epigenome (Fig. 3). This technology can reactivate a silenced tumor suppressor or downregulate an overactive oncogenic pathway [37, 38]. While these gene-based therapies face delivery challenges in vivo, nanotechnology is a synergistic solution: nanoformulated vectors, including lipid nanoparticles and polymers, can transport genetic cargo directly into tumor sites and even codeliver gene therapies alongside conventional drugs, providing a multifaceted attack against resistance [34, 39–41].

Overcoming cancer resistance requires an integrated understanding of cellular signaling, epigenetic plasticity, and tumor microenvironment dynamics. By leveraging nanotechnology to modulate the TME and enhance drug/gene delivery and deploying gene therapy to resensitize cancer cells through genetic or epigenetic modifications, researchers are developing innovative treatments that preliminary evidence suggests could disrupt resistance mechanisms (Fig. 3) though these remain aspirational therapeutic goals supported primarily by preclinical evidence rather than established clinical outcomes. This review provides five unique contributions that distinguish it from existing literature on chemoresistance. First, we present an integrated mechanistic framework that systematically connects TME signals, epigenetic reprogramming, and resistance phenotypes as a unified axis, rather than treating these as isolated topics. Second, we emphasize the central role of microRNAs (miRNAs) as bidirectional integrators that link signaling pathways with epigenetic machinery through self-reinforcing circuits; a perspective not comprehensively addressed in previous reviews. Third, we demonstrate how the distinctive properties of the TME (hypoxia, acidosis, stromal interactions) create specific vulnerabilities that can be exploited through mechanism-based nanotechnology design, moving beyond generic nanocarrier discussions to rational therapeutic strategies. Fourth, we critically evaluate clinical translation challenges rather than presenting nanotechnology and gene therapy as established solutions, explicitly addressing limitations including EPR effect heterogeneity, delivery barriers, and biomarker validation gaps. Fifth, we provide an actionable framework for designing next-generation combination therapies that target multiple nodes within the TME-epigenetic axis simultaneously. This integrated perspective is essential for developing therapeutic strategies that address the complexity and adaptability of chemoresistance mechanisms.

### The evolution of chemotherapy and chemoresistance

The history of chemotherapy is one of remarkable scientific ingenuity, beginning with wartime observations and culminating in molecularly guided precision medicine. Paul Ehrlich’s early 20th-century vision of “magic bullets” laid the conceptual groundwork for chemotherapy [42]. However, a serendipitous discovery during World War II, when mustard gas exposure revealed profound bone marrow suppression, catalyzed the clinical use of nitrogen mustard derivatives such as mustine for lymphoma treatment in 1942 [43]. Sidney Farber’s subsequent demonstration that antifolates such as aminopterin could induce remission in pediatric acute lymphoblastic leukemia (ALL) marked the first pharmacological success against hematologic malignancies [44]. These foundational discoveries led to the development of cytotoxic agents, including methotrexate, 6-mercaptopurine, and vincristine, which have become pillars of pediatric oncology [45]. The 1960 s and 1970 s ushered in combination chemotherapy, with regimens such as POMP and MOPP achieving curative outcomes in patients with ALL and Hodgkin’s lymphoma [46–48]. The use of platinum-based agents, such as cisplatin and carboplatin, followed in the 1980 s, expanding the reach of chemotherapy to solid tumors. The turn of the millennium introduced targeted therapies and nanomedicine, exemplified by imatinib and liposomal doxorubicin, which offered improved specificity and tolerability [12, 49]. However, as chemotherapy has advanced, so have biological countermeasures of cancer cells.

**Fig. 1:**
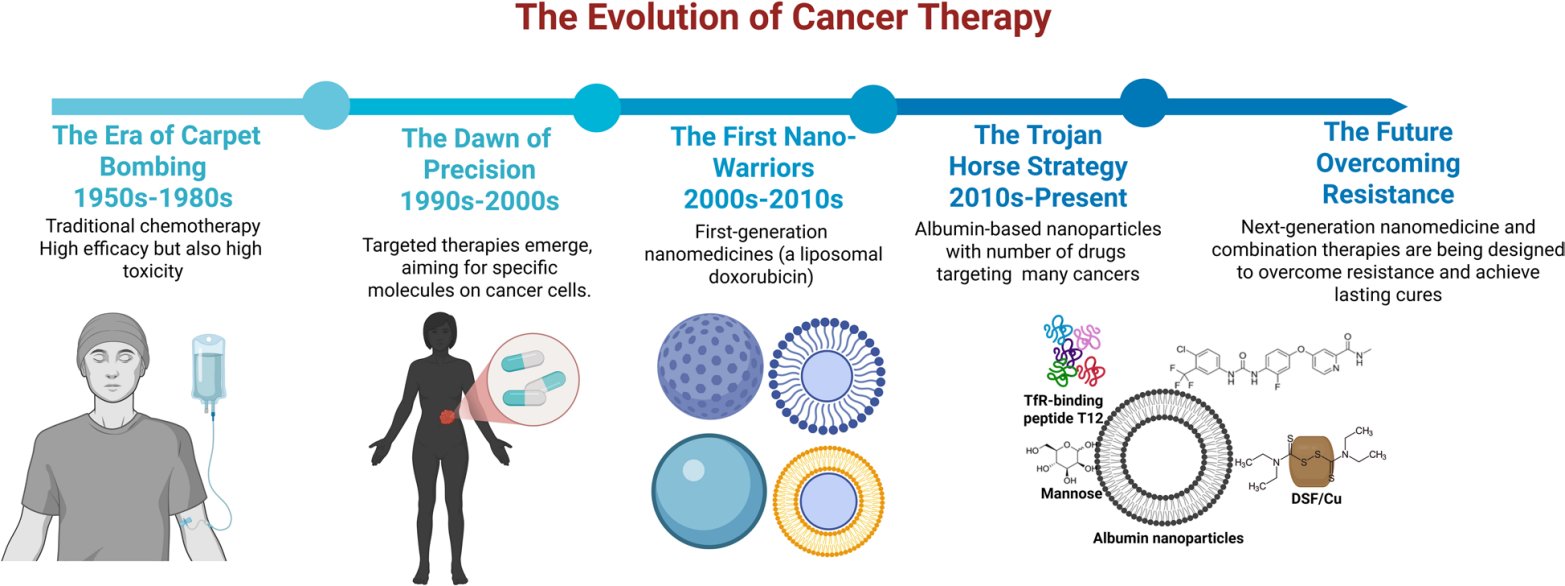
The Evolution of Cancer Therapy. This timeline depicts the major milestones in the development of cancer treatment strategies. It begins with the "Era of Carpet Bombing" (1950s–1980s), when traditional chemotherapy offered high efficacy but was associated with significant toxicity. The "Dawn of Precision" (1990s–2000s) marked the introduction of targeted therapies designed for specific molecules on cancer cells, improving selectivity and reducing side effects. In the "First Nano warriors" era (2000s–2010s), first-generation nanomedicines such as liposomal doxorubicin emerged, enhancing drug delivery. The "Trojan horse strategy" (2010s–present) represents the use of albumin-based nanoparticles carrying multiple drugs for more effective targeting of various cancers. Looking ahead, "The Future: Overcoming Resistance" highlights next-generation nanomedicine and combination therapies being developed to address therapy resistance and achieve lasting cures. Created in BioRender. Sharma, P. (2025) https://BioRender.com/9xyww15

Chemoresistance emerged as a formidable challenge, first noted in the 1950 s, when tumors that initially responded to treatment relapsed with diminished sensitivity [50, 51]. The discovery of multidrug resistance (MDR) in the 1970 s, driven by efflux pumps such as P-glycoprotein (P-gp), revealed that cancer cells can actively expel chemotherapeutic agents [52]. Over time, additional mechanisms, including enhanced DNA repair, altered drug metabolism, and evasion of apoptosis, have been revealed [53]. The tumor microenvironment (TME) plays a critical role, with stromal and immune cells secreting survival signals that shield cancer cells from cytotoxic stress [54]. In the 2000 s, epigenetic regulation and cellular plasticity emerged as key contributors to resistance. Processes such as epithelial-to-mesenchymal transition (EMT) and the persistence of cancer stem cells (CSCs) are linked to both metastasis and therapeutic failure [55]. Currently, chemoresistance is understood as a dynamic, multifactorial process. Strategies to overcome resistance include nanoparticle-based drug delivery, epigenetic modulators, and combination regimens designed to prevent adaptive resistance [56–58]. The intertwined histories of chemotherapy and chemoresistance underscore the need for integrative, adaptive approaches to achieve durable cancer control (Fig. 1).

## Cellular signaling & epigenetics in chemoresistance

Cancer cells frequently activate prosurvival and antiapoptotic signaling pathways that blunt the effects of chemotherapy. The classic examples include the PI3K/AKT/mTOR pathways, which promote cell growth and survival while inhibiting apoptosis. Hyperactivation of PI3K/AKT via PTEN loss or PIK3CA mutation is strongly linked to drug resistance across cancers [11, 14, 59]. Increased AKT signaling is correlated with resistance to paclitaxel and cisplatin in ovarian cancer and to doxorubicin in gastric cancer, partly through the upregulation of antiapoptotic proteins such as Bcl2 and MCL1 and inactivation of proapoptotic factors [60]. The MAPK/ERK pathway (Ras/Raf/MEK/ERK cascade) drives proliferation; its reactivation is a common escape mechanism after targeted therapies and can mediate resistance to cytotoxic drugs by enhancing DNA repair and cell cycle progression [59, 61, 62]. For example, MAPK signaling contributes to the tolerability of DNA damage, and elevated ERK activity has been implicated in cisplatin and vincristine resistance in certain tumors. The NF-κB pathway is another central player that is a stress-responsive transcription factor that induces genes involved in cell survival (cMyc, Cyclin D1), drug efflux pumps, and cytokines. The constitutive NF-κB activation observed in many resistant cancers makes cells more refractory to apoptosis and promotes a chronic inflammatory milieu that can further support resistance [63–66]. Additionally, Wnt/β-catenin activation can drive the expression of stemness genes and drug efflux transporters such as ABCB1, whereas Notch signaling in tumor cells often upregulates survival pathways and is linked to resistance to platinum and taxanes [1, 67]. Indeed, the overexpression of Wnt target genes was observed in cisplatin-resistant bladder cancer, which was mediated by an upregulated lncRNA (UCA1) that promoted Wnt signaling and cell survival. Additionally, pathways such as the TGFβ/SMAD, JAK/STAT3, and Hedgehog pathways contribute to resistance by inducing EMT and an immunosuppressive, fibrotic TME; for example, TGFβ can induce EMT transcription factors and cause tumors to become more invasive and chemotolerant [68].

### Epigenetic mechanisms of resistance

**Fig. 2:**
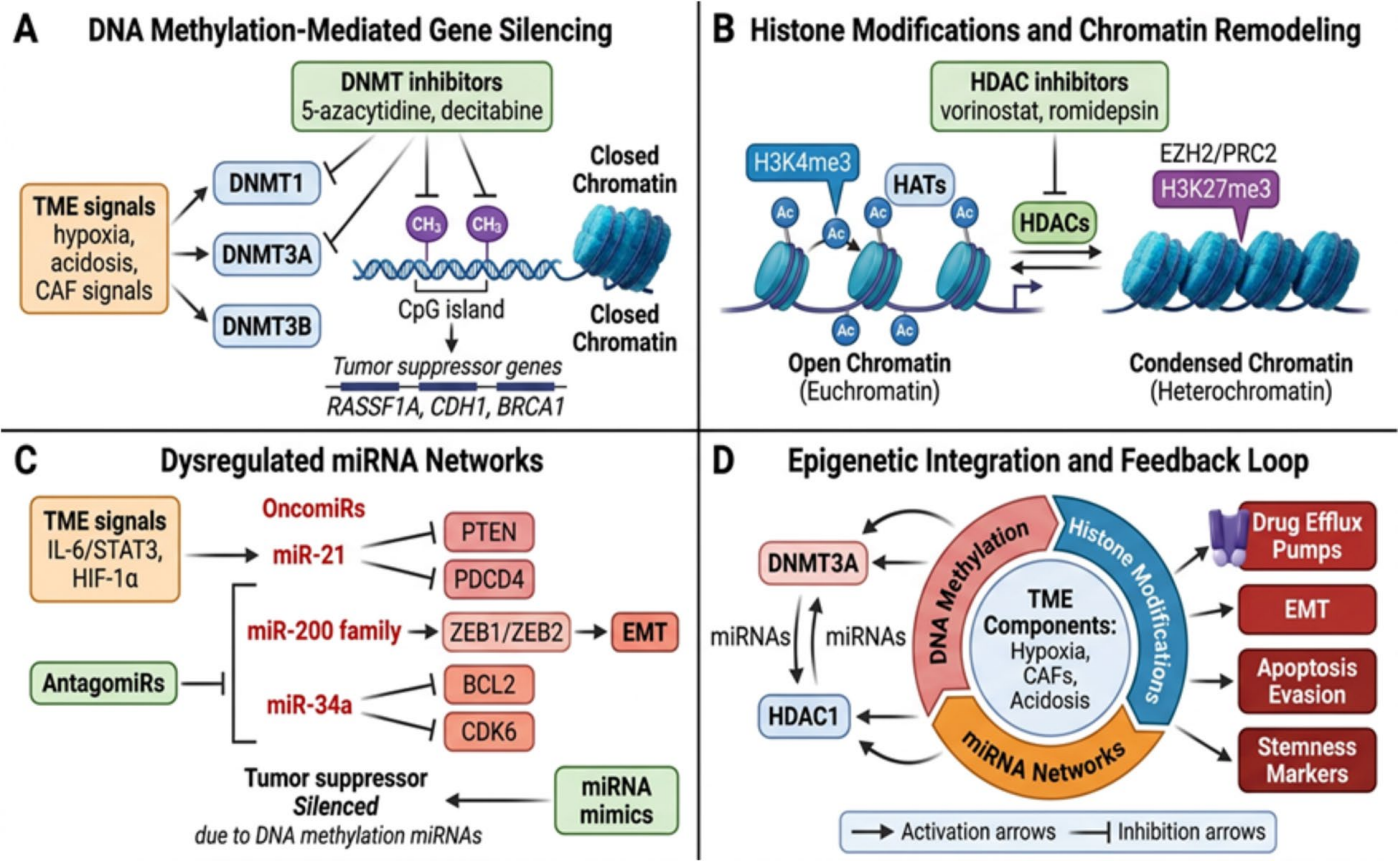
Epigenetic mechanisms driving chemoresistance in cancer. **A** DNA methylation–mediated gene silencing. Tumor microenvironment (TME) cues, including hypoxia, acidosis, and cancer-associated fibroblast (CAF) signaling, activate DNMT1, DNMT3A, and DNMT3B, which methylate CpG islands in the promoters of tumor suppressor genes such as RASSF1A, CDH1, and BRCA1, leading to chromatin compaction and transcriptional silencing. DNMT inhibitors, including 5-azacytidine and decitabine, can reverse this process. **B** Histone modifications and chromatin remodeling. The balance between histone acetyltransferase-driven chromatin opening and HDAC/EZH2–PRC2-mediated chromatin condensation, marked by H3K27me3, determines the transcriptional state of resistance-associated genes. HDAC inhibitors, such as vorinostat and romidepsin, restore histone acetylation and reactivate silenced loci. **C** Dysregulated miRNA networks. TME-associated signals, including IL-6/STAT3 and HIF-1α, induce oncogenic miRNAs such as miR-21, miR-200 family members, and miR-34a, while tumor-suppressive miRNAs are suppressed by DNA methylation. AntagomiRs and miRNA mimics may restore this regulatory balance. **D** Epigenetic integration and feedback loops. Hypoxia, CAFs, and acidosis reinforce interconnected DNA methylation, histone modification, and miRNA programs that promote drug efflux, EMT, apoptosis evasion, and stemness-associated phenotypes. Created in BioRender. Sharma, P. Created in BioRender. Sharma, P. https://BioRender.com/9uros8d

In addition to genetic mutations, stable changes in gene expression driven by epigenetic alterations are increasingly recognized as drivers of chemoresistance [1, 69]. These epigenetic changes include DNA methylation (often resulting in gene silencing when it occurs at promoter CpG islands), diverse histone modifications (such as acetylation and methylation, which can remodel chromatin structure), and the regulatory effects of noncoding RNAs, including microRNAs and long noncoding RNAs. These changes do not alter the DNA sequence but can heritably turn genes on or off, enabling cancer cells to adapt to drug pressure (Fig. 2). For example, resistant cancer cells often exhibit promoter hypermethylation of tumor suppressor genes or DNA repair genes, leading to their silencing. In ovarian cancer, acquired cisplatin resistance has been linked to hypermethylation-mediated repression of genes in cell adhesion and DNA damage pathways, alongside hypomethylation (loss of methylation) at oncogenic pathways such as the PI3K/AKT and TGFβ pathways, thereby activating survival signals [70]. This shift in the methylation pattern silences cell cycle checkpoints and activates growth pathways, illustrating how epigenetic reprogramming can endow cancer cells with a survival advantage under chemotherapy. Similarly, modifications of histones can alter the expression of large gene sets; increased histone deacetylation (via upregulation of HDACs) in tumors generally compacts chromatin and silences proapoptotic genes, contributing to drug tolerance. The overexpression of specific histone methyltransferases or demethylases can also drive resistance by repressing tumor suppressors or activating oncogenes. A recent comprehensive review highlighted that aberrant epigenetic regulation, including DNA hypermethylation, histone deacetylation, and chromatin remodeling, is common in resistant cancers and that targeting these epigenetic enzymes can restore drug sensitivity [38]. Notably, histone demethylases such as LSD1 and the JmjC family are frequently upregulated in resistant states and sustain the expression of genes involved in stemness and EMT; the inhibition or knockdown of these demethylases has been shown to resensitize tumors to therapeutic approaches.

Noncoding RNAs provide an additional layer of epigenetic regulation in cancer drug resistance. MicroRNAs (miRNAs) of ~ 22 nucleotides can post transcriptionally downregulate target mRNAs, and their dysregulation is linked to chemoresistance [1, 71, 72]. For example, miR-499a is markedly overexpressed in drug-resistant cervical cancer cells, where it directly downregulates the SOX6 gene, resulting in increased proliferation, reduced apoptosis, and resistance to chemotherapy [73]. In contrast, the loss of certain tumor suppressor miRNAs (through epigenetic silencing) can lead to the overexpression of oncogenic proteins. Long noncoding RNAs (lncRNAs) (> 200 nucleotides) also modulate gene expression at the transcriptional and epigenetic levels [1, 74]. The lncRNA UCA1 mentioned above is one example: upregulation of UCA1 in cisplatin-resistant cells leads to activation of Wnt signaling via increased Wnt6 expression, thereby promoting cell survival and drug resistance. LncRNAs can recruit chromatin-modifying complexes to specific genes, creating localized epigenetic changes that favor resistance. Together, these epigenetic alterations, DNA methylation, histone modifications, and changes in miRNA/lncRNA expression can orchestrate a broad “reprogramming” of cancer cell fate. These changes often result in the enrichment of a drug-tolerant subpopulation of cancer cells with stem cell-like features and slow proliferation, making them less susceptible to drugs dependent on the cell cycle. More importantly, most epigenetic changes are reversible and can be targeted therapeutically. The concept of using epigenetic therapies (DNMT inhibitors, HDAC inhibitors, etc.) to reverse chemoresistance has gained traction, with clinical evidence that such agents can resensitize tumors [75].

### Crosstalk between signaling and epigenetic reprogramming

Cellular signaling and epigenetic regulation are not independent modalities; they interact in a bidirectional, self-reinforcing manner, especially in the context of therapy resistance [10]. Chronic activation of oncogenic pathways can induce epigenetic modifications that lock cells into a resistant state. For example, the NF-κB and STAT3 pathways, which are activated by inflammatory cytokines such as IL-6 and TNFα from the tumor microenvironment, can recruit chromatin-modifying enzymes to the promoters of survival genes, thereby sustaining their high expression. In inflammatory breast cancer models, NF-κB signaling has been shown to cause hyperphosphorylation of the estrogen receptor along with overexpression of Cyclin D1 and c-Myc, changes that are partly maintained by epigenetic mechanisms that support tumor cell survival and proliferation [63]. EMT-inducing transcription factors (EMT-TFs), such as Snail, Slug, ZEB1/2, and TWIST, are activated by signaling pathways, including the TGFβ and Wnt pathways. These factors not only repress epithelial genes such as E-cadherin but also recruit histone-modifying enzymes such as histone deacetylases (HDACs) to permanently silence these genes. The epithelial‒mesenchymal transition (EMT) process is tightly regulated by epigenetic reprogramming, where EMT-TFs such as Snail cooperate with epigenetic regulators, including polycomb repressive complexes and LSD1, to establish repressive chromatin marks on epithelial gene promoters, thereby enforcing gene silencing and promoting EMT progression [76, 77].

These changes yield mesenchymal, stem-like cells that are inherently more drug resistant. Even if the original EMT-inducing signal (e.g., TGFβ) is removed, epigenetic marks can persist, maintaining the resistant phenotype. Conversely, epigenetic alterations can drive aberrant signaling. For example, DNA methylation-mediated silencing of negative regulators or phosphatases leads to unchecked pathway activity. Hypermethylation of tumor suppressor genes that normally restrain signaling pathways such as the Wnt or Notch pathways keeps these pathways constitutively active, promoting continuous proliferation and drug resistance. In platinum-resistant ovarian cancer, researchers have observed hypomethylation (demethylation) of promoters in genes of the PI3K/AKT pathway, suggesting that the loss of epigenetic repression contributes to sustained pathway activation and drug resistance. Similarly, epigenetic loss of PTEN or demethylation-driven upregulation of IGF1R can hyperactivate the PI3K/AKT and MAPK pathways, respectively, further enhancing resistance to therapy [59, 78, 79].

Evidence for feedback loops in cancer signaling is exemplified by Wnt/β-catenin activation, which can induce the expression of specific microRNAs or long noncoding RNAs that further enhance Wnt signaling or repress its inhibitors, establishing a positive feedback circuit. The epithelial‒mesenchymal transition (EMT) and cancer stem cell (CSC) programs provide prime examples of signaling–epigenetic crosstalk. Activation of EMT-associated pathways, including the TGF-β, Wnt, and Notch pathways, induces epigenetic alterations that confer stem-like properties. Conversely, cells harboring epigenetic changes, such as loss of H3K4 methylation at differentiation gene loci, frequently revert to a stem-like state and reactivate the same signaling pathways [80, 81]. This bidirectional interplay suggests that effective therapeutic strategies should simultaneously target both signaling networks and the underlying epigenetic mechanisms to prevent rapid adaptation. Collectively, chemoresistance arises from an interconnected network of signaling and chromatin changes, highlighting the need for interventions that combine pathway inhibition with the modulation of epigenetic regulators.

### Tumor microenvironment (TME) and chemoresistance

Drug resistance in cancer is not solely a tumor cell autonomous trait; it is profoundly influenced by the surrounding tumor microenvironment. The TME consists of a diverse array of cellular components, such as cancer-associated fibroblasts (CAFs), immune cells, and endothelial cells, and noncellular components, such as the extracellular matrix (ECM), blood vessels, oxygen, nutrient gradients, and soluble factors (cytokines and growth factors) [6, 19]. Together, these elements create a protective niche that can shield cancer cells from chemotherapy and even actively induce a more resistant phenotype. It is now understood that “to survive, cancer cells keep their enemies closer, meaning cancer cells interact closely with TME components such as stromal cells to gain survival advantages under therapy [69]. Below, we discuss four key TME factors, namely, hypoxia, acidosis, immune evasion/inflammation, and ECM stiffness, and how they contribute to chemoresistance and epigenetic plasticity in tumors (Fig. 2).

### Hypoxia

Rapid tumor growth often involves the development of functional blood vessels, leading to areas of low oxygen tension (hypoxia) in most solid tumors. Hypoxia is a hallmark of the TME that has major implications for therapy response. Oxygen deficiency stabilizes the transcription factor HIF-1α (hypoxia inducible factor 1), which in turn activates a broad genetic program enabling cells to adapt to low oxygen, including angiogenesis via VEGF, metabolic rewiring to anaerobic glycolysis, and pH regulation [82]. Unfortunately, these adaptations also promote malignancy and treatment resistance. Under hypoxic conditions, many chemotherapeutic agents lose efficacy; for example, the cytotoxicity of doxorubicin, cisplatin, and other drugs is reduced in hypoxic tumors. There are multiple reasons for the reduced drug potency (Fig. 2). First, some drugs rely on oxygen to generate reactive oxygen species or DNA damage, which can occur; the effects of radiation therapy are oxygen dependent, and hypoxic cells are more radioresistant than regular cancer cells are [83]. Second, hypoxia can cause cancer cells to enter a quiescent or slow cycling state (since proliferation is oxygen intensive), rendering them less susceptible to cell cycle-specific drugs such as 5FU or paclitaxel [84]. Third, HIF-1 activation upregulates the expression of drug efflux transporters such as P-glycoproteins) and antiapoptotic proteins, directly increasing chemoresistance. Hypoxia also selects for cells with EMT and stemlike properties, and HIF signaling can induce EMT transcription factors (Twist, Snail) and promote the maintenance of cancer stem cells, both of which are resistant to conventional therapy [84]. At the epigenetic level, hypoxia is known to alter the activity of enzymes such as histone demethylases and DNA methyltransferases (many of which require oxygen and αketoglutarate to function). Hypoxic tumors often exhibit a more undifferentiated, epigenetically plastic state. For example, HIF-1 can recruit corepressors and histone modifiers (such as HDACs) to certain gene loci [85], and global DNA hypermethylation has been observed as a response to chronic hypoxia, which silences differentiation genes and reinforces a stem cell-like, resistant phenotype. Thus, hypoxia not only physically creates zones of drug inactivity but also actively triggers molecular changes in cancer cells that underlie chemoresistance. Clinically, tumor hypoxia is correlated with poor outcomes and therapeutic failure in many cancers. Accordingly, strategies to counteract hypoxia or exploit it are being pursued, for example, delivering oxygen carriers or designing hypoxia-activated prodrugs that cytotoxin in reduced O₂ areas.

### Acidosis (low-pH microenvironment)

In conjunction with hypoxia, tumors commonly exhibit an acidic extracellular pH ranging from 6.5–6.9, in contrast to the near-neutral pH of approximately 7.4 found in normal tissues. Owing to the Warburg effect, the preference of cancer cells for glycolysis even in the presence of reduced oxygen leads to excess lactic acid production [86]. Additionally, poor perfusion also causes the buildup of CO₂ and protons. This acidic TME has several consequences that hinder chemotherapy. This acidic microenvironment adversely affects chemotherapy by altering drug properties; many drugs, particularly weakly basic agents, become protonated at low pH, which reduces their membrane permeability and cellular uptake. For example, doxorubicin tends to be sequestered within acidic intracellular vesicles and actively effluxes from cancer cells rather than being transported to the nucleus when exposed to acidic extracellular pH. Additionally, some chemotherapeutic agents degrade or lose activity in acidic environments [87]. In addition to direct effects on drugs, acidosis triggers cellular stress responses that confer resistance. Under chronic low pH, tumor cells may upregulate pH regulators such as carbonic anhydrase IX, proton pumps and stress tolerance pathways such as autophagy, a process that can help cells survive chemotherapy by recycling damaged components. Acidosis has been shown to increase the expression of multidrug resistance proteins and heat shock proteins that protect tumor cells. Notably, an acidic environment can alter epigenetic states; for example, acidosis can lead to global histone modifications. A novel epigenetic marker, lysine lactylation of histones, was recently discovered. Derived from lactic acid, it can directly modify histones and affect gene expression in macrophages and potentially tumor cells, linking metabolism to epigenetic gene regulation [88]. Moreover, lactic acid can inhibit histone deacetylases, leading to hyperacetylation of certain histones and aberrant gene expression in tumor cells. The net result is epigenetic plasticity, which may allow some cells to turn on survival pathways [89]. Acidosis also affects the behavior of immune cells: low pH can impair cytotoxic T-cell and NK cell function, indirectly aiding tumor survival during treatment by limiting immune-mediated clearance [90]. Overall, the acidic TME promotes tumor aggressiveness and drug tolerance. As one review succinctly noted, tumor acidity “enhances the tolerance of tumor cells to a wide range of chemotherapeutic agents” by reducing drug efficacy and fostering resilient cell states. Therapeutically, this has prompted interest in pH-responsive drug delivery and buffering strategies. Agents that increase tumor pH (such as bicarbonate) or nanocarriers that exploit low pH to trigger drug release specifically in tumors are being explored to overcome acidosis-driven resistance.

### Immune evasion and inflammation

The immune microenvironment of tumors, sometimes called the tumor immune microenvironment (TIME), profoundly affects the response to therapy. An immunosuppressive, “cold” tumor (with few cytotoxic T cells and a predominance of suppressive cells) tends to be more chemoresistant than an immune-active “hot” tumor, which can undergo immune-mediated cell death in combination with chemotherapy (Fig. 2). Tumor-associated macrophages (TAMs), often of an M2-like, protumor phenotype, are particularly implicated in chemoresistance [91, 92]. TAMs secrete a plethora of cytokines (IL6, IL10, TNFα, and TGFβ) and growth factors that activate survival pathways in cancer cells. For example, IL6 from TAMs can activate the JAK/STAT3 pathway in tumor cells, increasing the levels of antiapoptotic proteins and DNA repair enzymes. In colorectal cancer, TAM-derived IL6 was shown to increase the resistance of tumor cells to oxaliplatin by reducing drug-induced apoptosis, neutralizing IL6, or depleting TAMs to restore chemosensitivity. Similarly, TNFα from macrophages activates NF-κB in cancer cells; NF-κB then induces targets such as cFLIP, BclxL, and Cyclin D1, which blunt the efficacy of chemotherapy [63]. A striking positive feedback loop has been observed in breast cancer, where TAM-secreted CCL2 chemokines activate PI3K/AKT in tumor cells, causing them to resist tamoxifen; in turn, resistant tumor cells produce TNFα, which further polarizes macrophages to the M2 state, where they secrete more CCL2 [93]. This loop is involved in endocrine resistance, illustrating how reciprocity between tumor and immune cells drives drug resistance. TAMs can also release exosomes containing microRNAs or proteins that transfer resistance traits (e.g., delivering miR21 to tumor cells to activate PI3K/AKT). In addition to macrophages, regulatory T cells (Tregs) and myeloid-derived suppressor cells (MDSCs) in the TME can secrete TGFβ and other factors that induce EMT and chemoresistant phenotypes. Chronic inflammation in the TME mediated by NF-κB, STAT3, and COX2/PGE2 signaling causes continuous activation of survival pathways and DNA damage responses, helping tumor cells survive chemotherapy that would otherwise kill them. In fact, NF-κB in the TME not only acts on cancer cells but also upregulates checkpoint ligands (such as PDL1) and proangiogenic factors, remodeling the environment to be more supportive of tumor regrowth after treatment [94]. Immune-mediated selection plays a critical role in therapeutic resistance during cancer treatment. Chemotherapy often eradicates more immunogenic tumor cell populations, resulting in the survival and expansion of tumor cells with diminished immune visibility, termed “immune-edited” cells. These immune-edited populations frequently coincide with intrinsically drug-resistant subsets, supporting a dual mechanism of resistance that combines immune evasion and chemoresistance. Additionally, the absence of immune engagement disrupts cooperative cell death pathways that typically amplify the overall therapeutic efficacy of treatment [95, 96]. However, dying tumor cells release antigens and danger-associated signals, which can initiate immune responses against residual malignant cells. In an immunosuppressed tumor microenvironment (TME), however, immune activation is largely absent, allowing surviving cells to persist and expand. Indeed, clinical data from patients with hormone receptor-positive/HER2-negative breast cancer show that higher tumor HLA class I expression predicts a pathologic complete response (pCR) to anthracycline/taxane neoadjuvant chemotherapy. This association reflects increased tumor-infiltrating lymphocytes (TILs), particularly CD8+ cytotoxic T cells, indicative of enhanced antitumor immunity in tumors with high HLA class I levels [97]. However, in patients receiving a combination of chemotherapy and bevacizumab, posttreatment residual tumors demonstrate fewer CD8+ T cells alongside increased expression of M2 macrophage-specific genes, signaling a shift toward an immunosuppressive tumor microenvironment that may contribute to therapeutic resistance. These findings underscore a complex immune dynamic in the treatment response, where HLA class I expression fosters immune-mediated tumor clearance during chemotherapy, but combination therapies can alter immune cell composition and potentially impair effective antitumor immunity [98]. Crosstalk between immune signaling and epigenetic regulation, such as prolonged cytokine exposure, such as IL-6, which can drive DNA methylation changes in tumor cells, is increasingly recognized. This leads to the silencing of the antigen-presenting machinery and facilitates immune evasion. Conversely, epigenetic agents such as DNA methyltransferase inhibitors can restore the immune visibility of tumors by inducing the expression of otherwise silenced immunogenic antigens [99]. In summary, immune evasion creates a sanctuary for tumor cells under therapy, while inflammatory signals actively promote resistance. Novel strategies are being employed to reprogram the immune TME (e.g., repolarizing TAMs to a tumoricidal M1 phenotype or using immune checkpoint inhibitors) that can synergize with chemotherapy to turn a “cold” tumor into a “hot” tumor, a drug-responsive one. Indeed, HDAC inhibitors and DNA methylation inhibitors have been shown to increase antigen presentation on the tumor cell surface, in turn increasing tumor immunogenicity for more effective immunotherapies. These strategies suggest that epigenetic modifiers can counteract immune-related resistance mechanisms.

### ECM stiffness and physical barriers

Solid tumors are characterized by an abnormal, desmoplastic stroma and a dense network of collagen, fibronectin, hyaluronan, and other matrix components produced by CAFs. This ECM not only acts as a physical barrier to drug penetration but also provides biochemical and mechanical signals to cancer cells that influence their drug response [100]. A stiff ECM (as found in breast cancer, pancreatic cancer, etc.) increases interstitial fluid pressure and solid stress, impeding the distribution of chemotherapy within the tumor [101, 102]. Large drugs or nanoparticles may not be effectively perfused beyond the tumor periphery in highly fibrotic tumors, resulting in surviving cells in poorly penetrated regions. However, beyond limiting delivery, mechanotransduction, the process by which cells sense and respond to mechanical forces, plays a significant role in chemoresistance. Cancer cells adhering to a stiff matrix or encountering high tissue tension activate signaling pathways such as integrin/FAK (focal adhesion kinase), Rho/ROCK, and downstream effectors such as YAP/TAZ, which are transcriptional coactivators in the Hippo pathway [9, 11, 103]. These mechano-signaling pathways converge on many of the same nodes as growth factor signaling does, promoting proliferation, survival, and stemness. For example, integrin engagement on a stiff ECM triggers FAK and PI3K/AKT signaling, leading to the upregulation of Survivin and other apoptosis inhibitors. In one study, culturing cancer cells on a fibronectin-coated stiff substrate led to activation of the PI3K/Akt2 Survivin axis and rendered docetaxel-resistant cells. Similarly, increased matrix stiffness in pancreatic cancer models caused the nuclear translocation of β-catenin and YAP, the induction of EMT (loss of E-cadherin, gain of vimentin), and, consequently, resistance to paclitaxel. Stiffness can also induce cell cycle arrest in the G₀/G₁ phase in some contexts as cells adapt, which can protect them from drugs targeting the S/G₂/M phase. Furthermore, adhesion to the ECM via integrins can initiate a specific resistance program known as CAMDR (cell adhesion-mediated drug resistance). Upon binding the ECM, tumor cells become less susceptible to anoikis and chemotherapy, partly by activating NF-κB and AKT and increasing drug efflux pump expression. For example, the adhesion of multiple myeloma cells to fibronectin via integrins confers resistance to melphalan and other drugs, a classic case of CAMDR. Similarly, in solid tumors, a stiff collagen-rich matrix can increase the expression of ABC transporters and antiapoptotic Bcl2 family proteins in cancer cells. A few recent reviews illustrated that growing breast cancer cells on a stiff hydrogel activated integrin FAK signaling, which inactivated the Hippo pathway, thereby activating YAP; active YAP then increased the expression of the drug efflux transporter ABCB1 (P-gp) and other survival genes, leading to doxorubicin resistance [11, 104]. In head and neck cancer stem cells, hyaluronic acid (HA)-rich matrices engage CD44 to promote stemness and therapy resistance via modulation of the miR-302 axis. Specifically, HA–CD44 interactions lead to complex formation with key stem cell transcription factors (Oct4, Sox2, and Nanog), which in turn upregulate miR-302 expression. This cascade suppresses epigenetic regulators (AOF1/AOF2/DNMT1) and induces survival proteins, facilitating self-renewal, clonal formation, and chemoresistance, such as resistance to cisplatin. The mechanistic role of CD44 in this pathway has been directly demonstrated in head and neck squamous cell carcinoma models, with miR-302 acting as a central mediator of HA CD44-driven stemness and resistance phenotypes [105]. Blockade of miR-302 disrupts these stem-like features and sensitizes cells to chemotherapy, highlighting the functional relevance of the pathway in therapeutic resistance. These findings underscore that ECM remodeling is not just a bystander effect but also a driver of chemoresistance. Epigenetically, mechanical signals can also influence chromatin YAP/TAZ, for example, by interacting with chromatin modifiers, and can induce epigenetic changes that support a stemlike, drug-resistant state. Stiffness-associated signals have been linked to increased histone acetylation at genes that promote cell survival, although this is an emerging area of research [106, 107]. From a therapeutic standpoint, approaches to soften the tumor stroma or inhibit mechanotransduction show promise. The enzymatic degradation of collagen (via collagenase or hyaluronidase, e.g., PEGPH20 in pancreatic cancer) can improve drug penetration and has shown improved chemotherapeutic efficacy in trials [108]. Inhibitors of FAK and YAP have also been tested for their ability to reverse mechano-induced resistance [109]. For example, blocking FAK increases the sensitivity of previously ECM-adherent ovarian cancer cells to taxanes in preclinical models [110]. Collectively, targeting the physical aspects of the TME, such as high interstitial pressure, dense ECM, and mechanosensitive signaling cascades in tandem with conventional therapies, is now recognized as a crucial adjunct strategy to overcome resistance and improve clinical outcomes.

### TME-induced epigenetic plasticity

Importantly, the TME factors described above do not act in isolation; they often induce epigenetic changes in tumor cells that stabilize the drug-resistant phenotype. Hypoxia, acidosis, and inflammatory cytokines can all alter the tumor cell epigenome. Chronic hypoxia, as noted, can cause global DNA methylation changes, and histone modifications are sometimes termed an “epigenetic footprint” of hypoxia [85]. For example, HIF-1 was shown to recruit HDACs to the promoter of E-cadherin, contributing to EMT via epigenetic silencing of E-cadherin. Inflammatory signals such as IL6/STAT3 can recruit DNA methyltransferases to promoters of tumor suppressor genes, silencing them and promoting a stemlike state [111]. Physical contact with stromal cells such as cancer cells that bind to fibroblasts or endothelial cells can transfer miRNAs or induce methylation changes, which is sometimes called “education” of cancer cells by the stroma. The plasticity imparted by epigenetic changes means that even if initial TME signals fluctuate, such as temporary reoxygenation and changes in immune cell infiltration, the tumor may not fully revert to a drug-sensitive state because epigenetic memory persists. The concept of “adaptive resistance” captures that initial stress (drug or TME stress) triggers reversible changes; if the stress persists, those changes are cemented by epigenetic mechanisms into stable resistance. For example, short-term TGFβ exposure causes reversible EMT, but long-term exposure leads to DNA methylation of epithelial gene promoters, making the EMT largely irreversible and permanently increasing the resistance of cells to drugs. The therapeutic implications of targeting TME-induced epigenetic changes are compelling approaches, and drugs such as DNA methylation inhibitors (decitabine, azacitidine) or HDAC inhibitors (vorinostat, romidepsin) can erase some of the epigenetic marks that the TME imprints on tumor cells, thus resensitizing them. In fact, a pioneering phase II trial added hydralazine (a demethylating agent) and valproate (an HDAC inhibitor) to chemotherapy in patients who had been progressing on chemotherapy; the combination achieved partial responses or stable disease in 80% of these refractory patients [75], regardless of the tumor type, strongly suggesting that epigenetic therapy can reverse the broad chemoresistant phenotype maintained by the TME and other factors. This will be discussed more in later sections. Ultimately, the tumor microenvironment creates a protective cocoon for cancer cell hypoxia, and acidosis directly impairs drug action and leads to the selection of hardy cells; immune cells and cytokines activate survival pathways, and the ECM and stromal architecture form a physical and signaling barrier. Each of these factors also affects the cancer cell’s genome and epigenome, pushing it into a drug-tolerant state. An effective anticancer strategy must therefore target not only cancer cells but also their microenvironment.

## Mechanisms of chemoresistance in tumors

Chemoresistance in tumors is a complex, multifactorial process that arises from both intrinsic changes within cancer cells and extrinsic influences from the TME. These mechanisms are highly interconnected, enabling tumor cells to evade the cytotoxic effects of chemotherapy and contributing to relapse and treatment failure [112–114].

### Drug efflux and transporters

The overexpressed ATP binding cassette (ABC) transporters P-glycoprotein/ABCB1, MRP1, and BCRP actively extrude chemotherapeutics from cancer cells, reducing intracellular drug accumulation. These cassettes also alter drug metabolism through the upregulation of detoxifying enzymes, further attenuating therapeutic efficacy, especially in combination treatments [114]. For example, P-glycoprotein (P-gp/ABCB1) remains a principal mechanism of multidrug resistance that actively exports a wide array of chemotherapeutics, including cisplatin, paclitaxel, vinblastine, and doxorubicin, out of cancer cells, resulting in subtherapeutic intracellular drug concentrations (Fig. 2). Recent studies have confirmed that P-gp-mediated resistance is prevalent in breast, lung, gastrointestinal, and other cancers. Research has explored inhibitor compounds, RNA-based suppression, and CRISPR-mediated knockout of P-gp as means to restore drug sensitivity [115–118].

### Enhanced DNA repair

Many chemoresistant tumors upregulate DNA repair pathways, such as nucleotide excision repair (NER) and homologous recombination, aiding in the rapid resolution of DNA damage caused by drugs such as platinum compounds and topoisomerase inhibitors (Fig. 2). The upregulation of DNA repair proteins such as ERCC1 and XRCC1 is closely tied to resistance, especially in lung, ovarian, and bladder cancer [114].

Upregulation of DNA repair pathways (base excision repair, nucleotide excision repair, homologous recombination) protects cells from chemotherapy-induced DNA damage. MGMT (O6-methylguanine-DNA methyltransferase) is demethylated in resistant gliomas, conferring temozolomide resistance [119, 120].

### Survival pathway activation

Key oncogenic signaling pathways, including the PI3K/AKT, MAPK/ERK, and NF-κB pathways, are frequently hyperactivated in resistant tumors. These networks drive the expression of antiapoptotic proteins (Bcl2, IAPs) and suppress the cell death machinery, fortifying cancer cells against drug-induced apoptosis. Wnt/β-catenin signaling also promotes cancer stemness and resistance, particularly through the upregulation of drug efflux genes and the maintenance of self-renewing cell populations [112, 113, 121]. A recent study revealed ion channel-dependent activation of AKT (via Orai/TRPC channels) to ABC transporter expression, revealing a novel intersection between signaling and the efflux machinery [122].

### Tumor Microenvironment Factors

The TME is a critical player in chemoresistance. Cancer-associated fibroblasts (CAFs) secrete growth factors (HGF, EGF) and cytokines (IL6, CXCL12/SDF1) that activate survival and antiapoptotic signals in tumor cells and promote epithelial mesenchymal transition (EMT). The rigid ECM can both sequester drugs and physically impede their delivery, particularly in solid tumors such as pancreatic cancer. Extracellular vehicles (EVs) facilitate the horizontal transfer of drug resistance factors, including P-gp and drug resistance–associated nucleic acids, between cells within the TME [116, 123]. Hypoxia stabilizes HIF-1α, inducing glycolytic reprogramming, angiogenesis, drug efflux, and antiapoptotic signaling [124]. Cancer-associated fibroblasts (CAFs) secrete growth factors (e.g., HGF, IL6, and TGFβ), chemokines (CXCL12), and ECM remodeling enzymes (MMPs), promoting survival signaling, physical drug exclusion, and CAMDR [125]. Immune suppression is orchestrated via TAMs, Tregs, and cytokine networks; exosome-mediated macrophage polarization (e.g., CD19^+^ PDL1^+^ macrophages) further inhibits antitumor immunity [126]. Tumor-associated immunosuppressive cells, mainly TAMs and Tregs, further enhance drug resistance by protecting cancer cells from immune clearance and releasing factors that promote survival and repair after chemotherapy [127].

#### Cancer stem cells

Cancer stem cells, a small subpopulation within tumors defined by markers such as CD44, CD133, and ALDH, exhibit high levels of ABC transporters and robust DNA repair capacity, allowing them to evade chemotherapy. Therapy-resistant CSCs exhibit quiescence, enhanced DNA repair, and efflux capacity, allowing them to survive cytotoxic stress and drive relapse [114, 128]. Chemotherapy itself can promote CSC-like phenotypes, increasing the risk of recurrence and metastasis. Targeting both bulk tumor cells and CSCs is increasingly recognized as essential for durable responses [117, 127, 129]. Processes such as epithelial mesenchymal transition (EMT), which are regulated by transcription factors such as Snail, ZEB1, and TWIST, further instill stemness and resistance even in non-CSCs.

#### Autophagy-mediated survival

Cancer cells can activate autophagy as a process of self-digestion to survive chemotherapeutic stress. This cytoprotective autophagy allows the clearance of damaged components, the mitigation of reactive oxygen species, and survival during periods of metabolic challenge. Recent research supports the combined use of autophagy inhibitors (e.g., chloroquine and liensinine) with chemotherapy to overcome resistance in breast and prostate cancers [130].

#### Epithelial mesenchymal transition

EMT induces a phenotypic switch in cancer cells toward motile, mesenchymal-like states characterized by increased drug efflux, suppression of apoptosis, and increased DNA repair. This process, driven by transcription factors such as Snail and ZEB1, is associated with therapy resistance and immune evasion. EMT markers are directly linked to poor chemotherapy outcomes in several cancer types [131, 132]. Chemoresistance in cancer involves an interconnected network of effector mechanisms ranging from genetic and metabolic rewiring to microenvironmental modulation and intercellular crosstalk. More advanced integrative multiomics approaches targeting genomics, epigenomics, transcriptomics, proteomics, and metabolomics are illuminating the dynamic spatiotemporal landscape of resistance and can lead to new therapeutic avenues.

## MicroRNA and TME-epigenetic-resistance circuits

MicroRNAs (miRNAs) function as master post-transcriptional regulators that integrate TME signals, coordinate epigenetic reprogramming, and drive chemoresistance phenotypes [133, 134]. Several miRNA families have been mechanistically validated as central nodes in these circuits. Hypoxic CAFs export exosomes depleted in miR-200b-3p, which upon uptake by tumor cells derepress HMGB3, activating β-catenin/c-Myc signaling and reducing 5-fluorouracil sensitivity in colorectal cancer models [134, 135]; more broadly, loss of the miR-200 family promotes EMT by derepressing ZEB1/ZEB2 and SNAIL, locking cells into mesenchymal, therapy-resistant states via TGF-β/SMAD-mediated transcriptional repression [134, 135]. Within the tumor immune microenvironment, the IL-6 → STAT3 → miR-21 axis creates a self-amplifying loop in which miR-21 targeting PTEN and PDCD4 enhances STAT3 activity and reinforces immunosuppressive, pro-survival signaling [134, 136]. miR-34a, a p53-regulated tumor suppressor frequently silenced by promoter DNA methylation in resistant tumors, suppresses CSC pathways (CD44, Bcl-2, Notch, Wnt/β-catenin) and has been restored using nanocarrier delivery systems to resensitize tumors to chemotherapy in preclinical models [137]. Hypoxia-induced miR-210, driven by HIF-1α binding to miRNA promoter hypoxia response elements, promotes metabolic reprogramming toward glycolysis by targeting mitochondrial metabolism and DNA repair genes, reducing apoptotic sensitivity under chemotherapy [138]. Additionally, metabolic acidosis drives histone lactylation (H3K18la), a recently characterized modification that activates oncogenic miRNA clusters while suppressing pro-apoptotic miRNAs, creating a lactate (lactylation) miRNA feedback loop that sustains glycolytic resistance [139].

The crosstalk between miRNAs and epigenetic machinery is bidirectional and self-reinforcing. miRNAs directly target epigenetic enzymes; miR-29 family members suppress DNMT3A/3B to reactivate silenced tumor suppressors, miR-449a targets HDAC1 to increase chromatin accessibility, and multiple miRNAs reduce EZH2-mediated H3K27me3 repression [140, 141]. Conversely, TME-driven epigenetic changes silence tumor-suppressor miRNAs through promoter methylation and repressive histone marks (H3K27me3, H3K9me3), while active marks (H3K4me3, H3K27ac) promote oncogenic miRNA expression [141]. Loss of tumor-suppressor miRNAs then derepresses the same epigenetic enzymes, consolidating resistant chromatin states and enabling durable phenotypic plasticity [134, 141]. Therapeutically, antagomiRs targeting oncogenic miRNAs (anti-miR-21, anti-miR-155) and synthetic mimics restoring tumor suppressors (miR-34a, miR-200 family) have demonstrated chemosensitization in preclinical models when delivered via lipid nanoparticles, polymeric carriers, or engineered extracellular vesicles [133, 142–144]. Restoration of the miR-214-3p/miR-199a-5p cluster via engineered small EVs downregulated TLR4, β-catenin, and the vesicle-trafficking protein YKT6, resensitizing platinum-resistant ovarian cancer to carboplatin [145]. Early-phase clinical trials, including miR-34a mimic MRX34 and anti-miR-122 miravirsen, have established proof-of-concept for systemic miRNA targeting, though delivery efficiency, immunogenicity, and off-target effects remain key translational challenges [133, 146, 147]. Within the broader nanotherapeutic framework, TME-responsive nanocarriers releasing miRNA payloads under hypoxia or acidosis, combined with epigenetic drugs or immunotherapy, represent a rational multi-node strategy to disrupt the self-reinforcing circuits that sustain chemoresistance [7, 142–144].

### Nanotechnology-based strategies

**Fig. 3:**
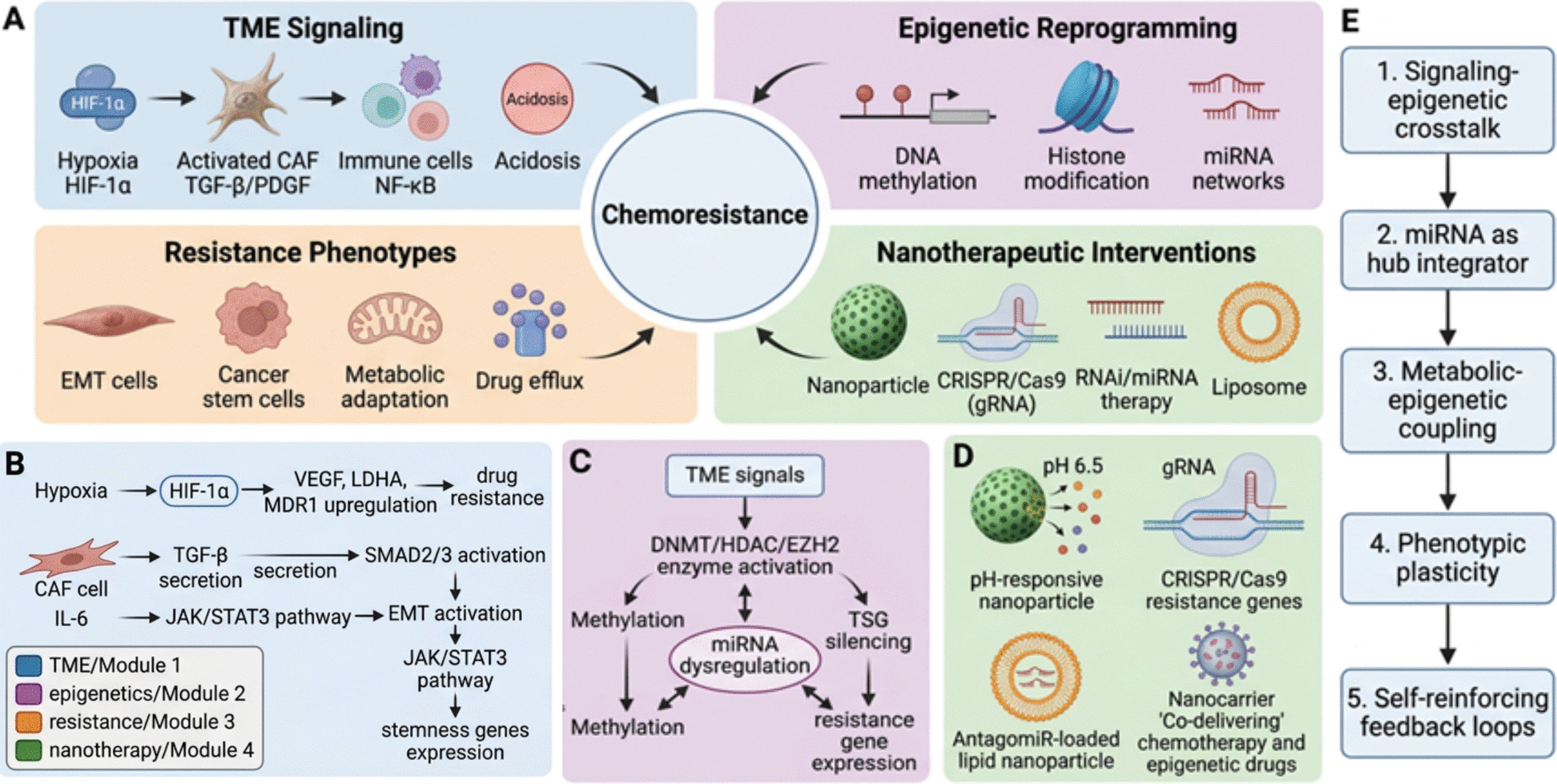
Integrated TME–epigenetic–nanotherapeutic axis in cancer chemoresistance. **A** Four interconnected determinants of chemoresistance: TME signaling (blue), epigenetic reprogramming (pink), resistance phenotypes (orange), and nanotherapeutic interventions (green), converging on chemoresistance. **B** Representative TME signaling cascades linking hypoxia, CAF-derived TGF-β, and IL-6 to HIF-1α, SMAD2/3, and JAK/STAT3 activation, respectively, with downstream induction of drug resistance, EMT, and stemness-associated programs. **C** Epigenetic reprogramming through activation of DNMT, HDAC, and EZH2, leading to aberrant DNA methylation, miRNA dysregulation, and silencing of tumour suppressor genes. **D** Representative nanotherapeutic strategies, including pH-responsive nanoparticles, CRISPR/Cas9-based targeting, antagomiR-loaded lipid nanoparticles, and co-delivery nanocarriers. **E** Integrated mechanistic model highlighting signalling–epigenetic crosstalk, miRNA-mediated integration, metabolic–epigenetic coupling, phenotypic plasticity, and self-reinforcing feedback loops. Created in BioRender. Sharma, P. https://BioRender.com/9uros8d

Nanotechnology has opened new avenues to overcome the barriers posed by the TME and to directly target resistant cancer cells. Nanoscale drug delivery systems (10–200 nm) can be engineered with defined functionalities: improving drug solubility, prolonging circulation, enhancing tumor accumulation via the enhanced permeability and retention (EPR) effect or active targeting, and enabling triggered release of payloads in response to TME cues. In chemoresistance, nanocarriers offer approaches that bypass or even exploit resistance mechanisms (Fig. 3).

#### Stimuli-responsive nanocarriers exploiting TME conditions

Given the hallmark features of the TME, namely, hypoxia, low pH, high reactive oxygen species (ROS), and upregulated enzymes, researchers have designed “smart” nanocarriers that, in vitro studies indicate, release their therapeutic cargo preferentially in tumor tissue by sensing these conditions (Fig. 3); mouse xenograft models show encouraging selectivity, though clinical translation of these stimuli-responsive systems warrants further investigation. For example, pH-responsive nanoparticles remain stable at the bloodstream pH (~ 7.4) but rapidly dissociate or change configuration in the acidic milieu of tumors (pH ~ 6.5), thereby dumping the drug at the tumor site. pH-sensitive liposomes or polymeric micelles can be formulated using materials that protonate or degrade at low pH. One notable design is a polymer–lipid hybrid nanoparticle with an acid‒labile bond attached to the drug: at acidic pH, the bond cleaves and releases the drug to achieve precise release in tumors and minimize systemic toxicity. Similarly, researchers have created polymeric nanogels or dendrimers that expand or disassemble at acidic pH values, thus increasing drug release or the exposure of targeting moieties only in tumors [148–150]. Hypoxia-responsive carriers constitute another exciting category. These exploit the reducing environment, and unique enzymes present under low O₂. For example, prodrugs can be attached to nanoparticles via hypoxia-cleavable linkers such as nitroimidazole and azobenzene groups, which are stable in oxygenated tissue but are cleaved by reductases in hypoxic zones. One example described in a previous study was that paclitaxel was linked to a quinone-containing group on a nanoparticle; in hypoxic tumor regions, cellular reductases reduce quinone, triggering the release of free paclitaxel selectively in the oxygen-deprived tumor core. This approach mitigates toxic effects on normal tissues and concentrates the drug at the hypoxic core where conventional therapies fail to target them [148, 149, 151, 152]. Some nanocarriers also carry their own oxygen supply, e.g., oxygen-loaded nanoparticles or perfluorocarbon-based oxygen carriers, which can locally release O₂ to alleviate hypoxia. Superparamagnetic iron oxide nanoparticles (SPIONs) have been used to transfer oxygen into tumors and simultaneously serve as drug carriers, thereby tackling hypoxia-induced resistance and enhancing therapies such as radiotherapy, which require oxygen [82, 153, 154]. Another common TME trigger is enzyme overexpression: tumors often have high levels of matrix metalloproteinases (MMPs), cathepsins, or hyaluronidases. NPs can be coated with a protective polymer or peptide that is cleaved by these enzymes, causing the NP to shed its coat and stick to cells or release drugs [155]. For example, an NP might have a stable PEG shell that is removed by MMP2 in the tumor, exposing cell-penetrating peptides that then drive the NP into cancer cells and improve deep tumor penetration and cellular uptake [156]. ROS-responsive nanoparticles have elevated ROS, which can be made of polymers containing thioketal or boronate bonds that are cleaved by ROS, triggering drug release inside cells where the level of ROS is high and resulting in the release of more drugs [157, 158]. By using these TME cues, stimuli-responsive nanocarriers achieve precision drug delivery, concentrating cytotoxic effects in tumors and reducing target exposure, which is especially beneficial when higher drug doses or combinations are needed to kill resistant cells. Crucially for chemoresistance, these smart nanocarriers can overcome several resistance mechanisms. For example, rapid intertumoral release can transiently create very high local drug concentrations that saturate efflux pumps such as P-gp and increase the pump capacity [159, 160]. Additionally, by codelivering drugs and releasing them simultaneously in the cell, one can ensure that combination therapies hit together before resistance factors are upregulated. Some pH-sensitive nanoparticles are also designed to invert charges in acidic environments, becoming positively charged to bypass endosomes, which facilitates the penetration of negatively charged tumor cell membranes and even disrupts endosomes to ensure that the drug escapes into the cytoso [161, 162] l. Such designs counter the problem of drugs becoming trapped due to low pH. Overall, stimulus-responsive nanomedicines aim to “turn the tumor’s defenses against itself” using the TME’s aberrant conditions as triggers to release more drug right where the tumor is most protected. Several of these designs are in preclinical development, and a few have entered clinical trials, such as pH-sensitive polymeric micelles with doxorubicin and hypoxia-activated prodrug nanoparticles.

#### Targeting cancer stem cells and stromal components

Cancer stem cells (CSCs) and other subpopulations, such as EMT-type cells, are major causes of chemoresistance and tumor relapse [163]. Nanotechnology offers ways to specifically target these cells. Cancer stem cells often express unique surface markers (e.g., CD44, CD133, EpCAM) and reside in niches (often hypoxic) [13]. Researchers have developed nanoparticles conjugated with ligands or antibodies for these markers to direct payloads to CSCs. For example, hyaluronic acid (HA)-decorated nanoparticles can target CD44, a receptor commonly overexpressed on CSCs. Yang et al. used HA-coated lipid–polymer nanoparticles carrying siRNA against MDR1 (P-gp) that homed to CD44^+^ cancer stem-like cells and silenced MDR1, restoring paclitaxel sensitivity and significantly inhibiting tumor growth in vivo in drug-resistant ovarian cancer [39]. By delivering siRNAs specifically to cells that drive resistance, the combination of nanoparticles and paclitaxel treatment overcomes established chemoresistance. This illustrates a broader theme of codelivery of a chemotherapeutic agent and a “resistance modulating” agent, such as a siRNA or inhibitor targeting a resistance gene, in one nanoparticle. New layer-by-layer (LbL) polymer nanoparticles have been used to coencapsulate doxorubicin with against Bcl2 and P-gp in a breast cancer model these NPs reduced MDR1 expression by 80% and reduced tumor burden. By packaging both agents together, all the resistant cells that get drug are simultaneously hit with the silencing RNA, maximizing re-sensitization. A study by Zhang et al., discussed this approach about tumor heterogeneity by making sure every nanoparticle act as a combination therapy “cocktail” on a single cell level [164].

Nanoparticles can be functionalized with mannose to target macrophage mannose receptors or other ligands to preferentially accumulate in TAMs [165, 166]. Delivery of drugs like bisphosphonates or PI3Kγ inhibitors to TAMs via NPs can reprogram or deplete TAMs, thereby removing their pro-survival influence on tumor cells [63, 92]. Another approach is targeting cancer associated fibroblasts (CAFs) which often express FAP (fibroblast activation protein) or PDGFRβ NPs carrying CAF inhibitory drugs or even cytotoxic can disrupt the stroma and improve overall drug delivery. A recent review highlighted progress in nanocarriers that specifically deliver drugs to CAFs, such as peptides that bind FAP on CAFs used to decorate nanoparticles, which reduced collagen production and decreased intertumoral pressure [167]. By “normalizing” the stroma or knocking down TGFβ in CAFs via siRNA NPs, these strategies help break the protective barrier and can prevent CAF induced resistance signals.

### Classes of nanoparticle systems

Several nanoparticle platforms have been explored for overcoming chemoresistance:

#### Liposomes

Spherical vesicles with a lipid bilayer, liposomes were among the first drug nanocarriers and have clinically approved examples such as PEGylated liposomal doxorubicin, Doxil [168]. Liposomes naturally accumulate in tumors via EPR and can encapsulate hydrophobic drugs, reducing systemic toxicity [169]. In the context of resistance, liposomes can alter pharmacokinetics to avoid peak plasma levels that cause toxicity, allowing higher cumulative dosing [170]. Notably, liposomal doxorubicin has shown efficacy in platinum and doxorubicin-resistant ovarian cancer, partly because its altered delivery evades some resistance mechanisms for example liposomes can fuse with cancer cell membranes and release doxorubicin intracellularly, bypassing some P-gp efflux at the cell surface [171, 172]. New generation liposomes are being tuned to address specific resistances: e.g., thermosensitive liposomes that release drug when heated accumulated in tumor, then mild hyperthermia triggers local release, and pH-sensitive liposomes which destabilize in acidic endosomes to release drug by ensuring the drug is not trapped in vesicles [27, 173]. By functionalizing liposomes with targeting ligands (antibodies, peptides), one can direct them to cancer cells or even specific TME components. For example, liposomes decorated with an anti-CD133 antibody could deliver a drug specifically to CD133 expressing CSCs in a tumor, sparing the bulk tumor cells until the CSCs which is the root of resistance [117]. Additionally, liposomes can co-deliver combinations: there are liposomal formulations carrying both doxorubicin and verapamil (P-gp inhibitor), aiming to inhibit efflux and deliver doxorubicin together to MDR tumors.

#### Polymeric nanoparticles and dendrimers

Polymeric NPs made of PLGA, poly alkyl cyanoacrylate, etc. allow for controlled release and protection of drugs. PLGA nanoparticles have been used to co-deliver paclitaxel and TRIAP1siRNA (TRIAP1 is a mediator of drug resistance in some tumors) with success in re-sensitizing resistant ovarian cancer in models [174]. Dendrimers are highly branched, treelike polymers with a central core and branches, yielding a monodisperse nanoscale particle with many surfaces’ functional groups. Their well-defined structure and multivalency make them appealing for precision targeting [175]. Dendrimers (like PAMAM dendrimers) can be conjugated with drugs, targeting moieties, and even imaging agents simultaneously. They have a nanometer size and can penetrate tissues well. In chemo resistant contexts, dendrimers have been used to deliver antisense oligonucleotides to knock down Bcl2 in resistant leukemia, and to carry cisplatin analogues that are released inside the cells [176]. One interesting approach is dendrimer drug conjugates that release drug in response to an intracellular trigger for example a glutathione sensitive linker releasing drug in the cytosol where GSH is high [176]. Dendrimer surfaces can also be modified with peptides like RGD which target integrins in angiogenic vessels or some cancer cells to improve uptake. A recent study showed that a dendrimer functionalized with TAT peptide (cell penetrating peptide) could ferry doxorubicin into drug resistant breast cancer cells and bypass P-gp, achieving higher nuclear drug accumulation than free doxorubicin [177, 178]. PAMAM dendrimers are versatile nanocarriers that can be precisely engineered for targeted and controlled drug delivery in cancer treatment. Their ability to conjugate drugs, targeting ligands, and cell-penetrating peptides enhances selective uptake, improves intracellular drug release, and overcomes drug resistance. These properties not only increase therapeutic efficacy but also reduce side effects by sparing normal cells. Overall, PAMAM dendrimers represent a promising platform to advance more effective and safer cancer therapies.

#### Inorganic nanoparticles

Inorganic nanoparticles such as gold nanoparticles (AuNPs) are inert, easy to functionalize with thiol containing molecules, and can also be used for photothermal therapy. AuNPs are highly amenable to surface modification, allowing precise conjugation with anticancer drugs such as doxorubicin to facilitate direct tumor targeting and reduce off-target toxicity. Owing to their high atomic number (Z), gold nanoparticles are effective as radiation dose enhancers, thereby improving outcomes in radiotherapy by increasing localized energy deposition within tumors [179–181].

For chemoresistance, one approach is photothermal therapy (PTT), where gold NPs accumulate in tumors and then are excited by near-infrared light; their plasmonic resonance efficiently transduces photon energy into heat, ablating nearby cancer cells, including those resistant to chemotherapy. This process not only directly destroys drug-resistant cells but also disrupts the tumor vasculature and can sensitize the remaining tumor cells to chemotherapeutic agents, potentially overcoming chemoresistance. PTT has been shown to achieve rapid and highly localized ablation of tumor cells at relatively low laser powers, further minimizing collateral damage [182, 183]. AuNPs are frequently coated with stimuli-responsive polymers, including pH- or enzyme-sensitive layers, to enable controlled drug release analogous to that of polymeric nanoparticles. Additionally, gold nanoparticle platforms have been used for nucleic acid delivery; spherical nucleic acid (SNA)-dense assemblies of siRNAs or antisense oligonucleotides on gold nanoparticle cores have demonstrated remarkable cellular uptake and knockdown efficiency in tumor models. Importantly, SNAs targeting the BCL2L12 gene have shown safety and potential for brain tumor (glioblastoma) gene silencing in early-phase clinical trials (e.g., NCT03020017), presenting a powerful framework for modulating oncogene or drug resistance gene expression in vivo [184, 185]. In addition to gold, other inorganic nanoparticles, such as mesoporous silica nanoparticles (MSNs), offer high-capacity, spatially controlled drug delivery. MSNs can be engineered with “capped” pores that respond to external stimuli, enabling the sequential and synergistic delivery of drug combinations such as doxorubicin and HDAC inhibitors. Furthermore, MSNs have been utilized to modify the tumor microenvironment to deliver agents such as collagenase to break down extracellular matrix barriers and improve treatment efficacy. Overall, inorganic nanoparticles, particularly gold-based structures, represent versatile, multifunctional platforms with demonstrated value in multimodal cancer therapies, including drug delivery, photothermal therapy, radiosensitization, and genetic modulation.

#### Exosome-mimetic nanoparticles

Exosomes are natural nanosized vesicles (30–150 nm) secreted by cells and are involved in intercellular communication. By transferring miRNAs and proteins between cells, tumor-derived exosomes actually contribute to drug resistance [186, 187]. However, their efficient cell targeting ability and intrinsic biocompatibility make exosomes attractive as drug delivery vehicles. Researchers are isolating exosomes from immune cells or mesenchymal stem cells and loading them with chemotherapeutics or siRNAs to target tumors. For example, exosomes engineered to carry let7 miRNA, which is a tumor-suppressive miRNA, have been used to treat KRAS-mutant cancer in mice [188]. Instead of isolating exosomes, which is a laborious task, scientists use cell-derived membrane vesicles or synthetic liposomes with exosomal surface proteins to mimic their homing ability [189]. These exosome mimetics can be engineered to display specific surface molecules, such as integrins, enabling them to mimic the tissue-specific homing capabilities characteristic of natural exosomes. In breast cancer models, exosomes loaded with doxorubicin showed better penetration and greater cytotoxicity in resistant cell lines than liposomes did [190], possibly because exosomes naturally fuse with recipient cells effectively and can evade P-gp efflux by releasing drugs directly into the cytosol. Exosome-like polymeric nanoparticles, also referred to as cellular nanoporation-derived vesicles, have been explored as drug delivery vehicles; these vesicles are generated by forcing cells through microfabricated filters, which induces membrane disruption and the release of vesicles loaded with therapeutics. These bioinspired carriers are designed to harness the targeting capabilities of natural exosomes while enabling scalable manufacturing processes, making them promising platforms for delivering gene therapies such as CRISPR–Cas9 components or siRNA molecules.

To sum up, preclinical evidence suggests that nanotechnology offers a versatile toolkit of carriers that could potentially be engineered to overcome specific mechanisms of chemoresistance. These include enhancing drug accumulation in poorly accessible tumor regions, codelivering multiple agents to synergistically inhibit resistance pathways, and selectively targeting chemoresistant cell populations through strategies such as targeting tumor-associated macrophages or relieving hypoxia — though clinical validation of these strategies warrants further investigation. Many of these nanomedicine approaches have demonstrated improved therapeutic efficacy in preclinical models of multidrug-resistant cancers. However, clinical translation remains challenging owing to the complexity of nanoparticle design, which must be balanced with considerations of safety and manufacturing reproducibility. Despite these hurdles, several nanoformulations, such as liposomal chemotherapies and albumin-bound drugs such as nab-paclitaxel, have already been established in oncology, with next-generation smart nanomedicines poised to promote the sensitization of tumors previously refractory to treatment.

### Gene therapy approaches

Gene therapy in cancer aims to directly manipulate the genetic or epigenetic machinery of cancer cells or immune cells to counteract drug resistance [191]. Unlike small molecules, which often target protein function, gene therapy can eliminate or repair the root causes of resistance at the DNA/RNA level. Recent breakthroughs in genome editing and nucleic acid delivery have invigorated this field, offering tools to knock out resistance-conferring genes, restore tumor suppressors, or resensitize cells through epigenetic modulation. Here, we discuss three major gene therapy modalities, CRISPR-Cas9 genome editing, RNA interference (RNAi) and antisense oligonucleotides, and CRISPR-based epigenetic editing, along with the challenges of delivering these therapies into the TME (Fig. 3).

#### CRISPR-Cas9 genome editing

In the evolving field of gene therapy, researchers are now focusing on altering the epigenetic landscape, which refers to the layers of chemical regulation that control gene activity, rather than editing the underlying DNA sequence. A powerful tool for this purpose is CRISPR dCas9, a version of Cas9 that cannot cut DNA but can be directed to specific sites via guide RNAs. By fusing dCas9 with different epigenetic effector domains, scientists can dial genes up or down by modifying promoter or enhancer activity or even reworking DNA methylation patterns to switch genes on or off in targeted ways [192, 193]. This approach is intended to combat chemoresistance. To date, most applications have used CRISPR to knock out genes that fuel resistance. For example, targeting ABCB1 (also known as MDR1), the gene encoding the P glycoprotein drug efflux pump, has helped restore drug sensitivity in cancer cell lines (Fig. 3) [33]. Researchers have also knocked out genes such as BCL2, BCLxL, mutant p53, and DNA repair players such as POLB and PARP1, weakening cancer cell survival mechanisms and enhancing their response to chemotherapy. Other studies have targeted RRM1 to resensitize cells to gemcitabine and knocked out NRF2 in KEAP1-mutant lung cancers to reverse their metabolic defenses [33]. Another layer of control comes from CRISPRa (activation) and CRISPRi (interference). These strategies use dCas9 teamed up with either activating domains such as VP64 or repressive domains such as KRAB or DNA methyltransferases. In the context of chemoresistance, one could imagine using CRISPRa to reactivate a silenced tumor suppressor, such as *MLH1*, whose promoter is often methylated and repressed, especially in mismatch repair–deficient cancers [194]. Restoring such genes could renew sensitivity to DNA-damaging agents. Likewise, one might use an acetyltransferase fused to dCas9 to increase the expression of proapoptotic genes such as *NOXA* or *BIM*, encouraging cancer cells to self-destruct under chemotherapy pressure. Conversely, CRISPRi could be deployed to suppress oncogenes that are otherwise difficult to target with drugs, providing a reversible and more controlled way to modulate gene expression [16]. However, delivering a large protein such as dCas9 fused to an effector remains a hurdle. One approach is to deliver it as mRNA or DNA via nanoparticles or viruses. Another strategy is to split the system: deliver dCas9 as one component (possibly even stably via a gene therapy vector) and then deliver small guide RNAs via nanoparticles to direct it. The safety of epigenome editing is expected to be greater than that of nucleolytic editing because dCas9 has no nuclease activity, so off-target binding might not have lasting effects unless it brings the effector to the wrong place. Efforts are ongoing to make the effectors more precise (e.g., light activated or drug inducible) so that one can, say, activate a silenced gene only for a short window to sensitize to chemotherapy and then eliminate the activation to avoid potential tumor suppressor hyperactivity in normal cells. While these genetic edits are powerful, delivering CRISPR components in vivo is challenging. Compared with viral vectors, nonviral delivery methods such as lipid nanoparticles carrying Cas9 mRNA and sgRNA are commonly utilized because of their reduced immunogenicity. In 2021, Intellia Therapeutics and Regeneron reported the first demonstration of in vivo CRISPR genome editing in humans using lipid nanoparticles (LNPs) to deliver CRISPR–Cas9 components targeting a liver gene (TTR gene) in patients with transthyretin amyloidosis. This approach achieved dose-dependent and durable reductions in disease-causing protein levels, providing a proof of principle for systemic in vivo genome editing in patients [195]. For applications in solid tumors, local or tumor-selective delivery is necessary to avoid gene editing in healthy tissues. Nanotechnology plays a central role here; for example, polymeric nanoparticles have been engineered to deliver Cas9/sgRNA plasmids specifically to tumor tissue, enabling precise genome editing of cancer cells while minimizing off-target effects in normal organs [32, 196]. CRISPR technology is also being harnessed to engineer immune cells to overcome tumor resistance. In immunotherapy clinical trials, CRISPR/Cas9 has been used to disrupt the PD-1 gene in T cells, thereby preventing these cells from being inhibited by PD-L1 expressed on tumor cells. Notably, the first-in-human clinical trial of this approach was conducted in China, where PD-1 knockout T cells were infused into patients with advanced lung cancer. The results demonstrated that this strategy was feasible and safe, with gene-edited T cells persisting in patients and no significant off-target effects observed [197, 198]. While this approach is related primarily to immunotherapy rather than direct chemoresistance, it addresses immune evasion, a critical contributor to overall treatment failure. Similarly, CRISPR has been employed to engineer CAR-T cells lacking PD1 and endogenous T-cell receptors to minimize graft-versus-host disease, thereby increasing their efficacy against solid tumors [198]. The success of these trials shows that gene editing can be performed safely in humans; one can envision editing a patient’s tumor cells ex vivo to identify vulnerabilities or even delivering CRISPR in vivo to tumor sites. However, challenges such as potential off-target mutations (although these appear rare and low frequency for carefully designed guides [197] and efficient delivery to enough tumor cells exist. However, the precision of CRISPR in erasing specific resistance genes is unparalleled, and it could provide a more permanent solution than transient small molecule inhibition. For example, instead of continuous P-gp inhibition with a drug that affects normal tissues, CRISPR knockout of the MDR1 gene in the tumor could permanently eliminate drug efflux from those cells. This is still a young field, but early results are encouraging. A recent review by Rajanathadurai and colleagues highlighted the application of CRISPR-dCas9-based epigenetic effectors to reprogram cancer cell phenotypes. For example, dCas9 fused to the DNA demethylase TET1 was utilized in an epigenetic screening approach to identify methylated genes whose demethylation could resensitize resistant cancer cells to therapy [37]. In a recent study, researchers employed dCas9 fused to histone deacetylase 8 (HDAC8) to epigenetically target the ESR1 gene in endocrine-resistant breast cancer cells. This approach removes acetylation marks on histones, resulting in downregulation of ESR1 expression and restoring sensitivity to hormonal therapies such as tamoxifen [199]. Importantly, these molecular tools enable simultaneous modulation of multiple genes within their native regulatory contexts by using multiple guide RNAs, providing a degree of precision and control unattainable with traditional gene therapy approaches. What makes epigenetic editing particularly promising is its reversible nature: since the underlying DNA sequence remains unaltered, the induced epigenetic changes can potentially diminish over time or be modified again, offering a level of safety and flexibility not possible with permanent gene edits. This reversibility positions epigenetic editing as an attractive strategy for both fundamental research and future clinical applications.

#### RNAi and antisense oligonucleotides

RNA interference (RNAi) via small interfering RNAs (siRNAs) or microRNA mimics, as well as antisense oligonucleotides (ASOs), is used to silence gene expression at the mRNA level. These therapies do not alter the genome but can reduce the production of specific proteins that drive resistance as demonstrated in preclinical models. The potential advantage of these methods is that they are reversible and tunable, and multiple targets could potentially be addressed by combining different siRNAs; however, in vitro studies indicate that delivery efficiency and off-target effects remain important translational barriers (Fig. 3). Numerous studies have shown the potential of RNAi to reverse chemoresistance. For example, introducing MDR1-targeted siRNA in drug-resistant cancer cell lines dramatically reduces P-gp levels and restores the intracellular accumulation of chemotherapeutics [39, 200]. In xenograft models of resistant ovarian cancer, nanoparticle-delivered MDR1 siRNA combined with paclitaxel led to tumor regression, whereas paclitaxel alone had little effect [39]. Small interfering RNAs targeting antiapoptotic proteins such as BCL2, BCLxL, or Survivin (BIRC5) have been shown to lower the apoptotic threshold and sensitize tumors to chemotherapy. For example, systemically administered nanotherapeutic Bcl-2 siRNA inhibited tumor growth and enhanced the effects of chemotherapy in breast cancer models by promoting both autophagy and apoptosis [201]. In triple-negative breast cancer, targeting the oncomiR miR-21 with siRNA restored PTEN levels and increased doxorubicin-induced apoptosis in resistant cell lines [202]. In addition to antiapoptotic targets, RNA interference has also been used to disrupt survival signaling pathways. For example, siRNA-mediated silencing of EGFR in head and neck squamous carcinoma cells significantly reduced the IC₅₀ values for cisplatin, 5-fluorouracil, and docetaxel [203]. Similarly, in non-small cell lung cancer models, combining EGFR-specific siRNA with tyrosine kinase inhibitors or cetuximab further suppressed cell growth and triggered apoptosis even in tumors harboring resistance mutations [204]. Antisense oligonucleotides are short single-stranded DNA or RNA analogs that bind to a specific mRNA sequence and either promote its degradation through RNase H activity or block its translation into protein [205]. One of the earliest examples was oblimersen, also known as G3139, which was designed to target *BCL2* mRNA [206]. In clinical trials for melanoma and chronic lymphocytic leukemia, oblimersen was able to lower Bcl-2 protein levels and, when combined with chemotherapy, improved response rates in a subset of patients. Although it did not achieve strong results as a standalone treatment, antisense therapy could resensitize cancer cells to other drugs [207–210]. In recent years, newer antisense oligonucleotides with chemical modifications, such as locked nucleic acids, which make them more stable, more potent, and better able to bind their targets, have been developed. Several of these next-generation molecules are being tested against transcription factors associated with treatment resistance, including STAT3 and HIF-1α, with the aim of blocking key survival pathways in cancer cells [211, 212]. One of the first FDA-approved RNAi therapies for a genetic disease (not cancer), patisiran (siRNA for transthyretin), confirmed that systemic siRNA delivery is feasible in humans [213]. In cancer, a landmark trial by Tabernero et al. used ALNVSP, a lipid nanoparticle encapsulating two siRNAs (one for VEGF and one for KSP kinesin), in patients with liver-involved cancers [214]. The trial results were encouraging, as the therapy was safe, showed that RNAi-mediated target mRNA knockdown in tumor biopsies, and even achieved a complete response in an end-stage patient, the first clear demonstration of the ability of RNAi to help cancer patients. Although the ALNVSP has not progressed to market, it has paved the way for current clinical trials of siRNAs (e.g., siRNAs against β-catenin in liver tumors). Delivery of RNA remains a major challenge [215]. While viral vectors can deliver shRNA to induce RNAi, concerns over toxicity and immune reactions have shifted attention toward synthetic nanoparticles and ligand RNA conjugates, such as GalNAc, for targeted delivery to the liver [35, 216]. Clinical and preclinical studies have shown that nanoparticle-delivered siRNAs can safely and effectively decrease tumor growth and overcome resistance. Despite delivery challenges, advances in nanoparticle systems and chemical modifications are enhancing therapeutic potential. RNAi therapies represent a promising strategy to complement traditional chemotherapy and overcome multidrug resistance in cancer (Table 1).

**Table 1:** Terminated or discontinued clinical trials of nanocomposite-based therapies

NCT Number	Interventions	Study Status	Target tumor	Phase	Last Update Posted	Reason for Termination/Withdrawal	Critical Perspective
NCT03878524	Investigational* Armamentarium	Terminated	Various Advanced Cancers	Phase-I	3/4/2024	Low accrual	To understand why cancer drugs can stop working and how different cancers in different people respond to different types of therapy
NCT05494866	Cobicistat, Gemcitabine, Nab paclitaxel	Terminated	Advanced Stage or Metastatic Pancreatic Ductal Adenocarcinoma,	Phase-I	3/25/2025	Lack of efficacy paired with high toxicity	Suggests that metabolic inhibition (Cobicistat) cannot overcome established resistance without addressing the TME-driven survival signals
NCT04214093	AZD0466	Terminated	Advanced Solid Tumors, Lymphoma, Multiple Myeloma, Hematologic Malignancies	Phase-I	8/2/2023	Strategic change to clinical development plan	Dual inhibitor of Bcl-2 and Bcl-x linked to a PEGylated poly-L-lysine dendrimer
NCT05062980	TUSC2 tumor suppressor gene, PD-1 checkpoint inhibitor, Docetaxel	Terminated	Non-Small Cell Lung Cancer	Phase-I, phase-II	2/20/2025	Slow accrual/Competition	Dose-Escalation and Clinical Response Study of Quaratusugene Ozeplasmid in Combination with Pembrolizumab Versus Docetaxel with or Without Ramucirumab in Patients with Previously Treated Non-Small Cell Lung Cancer
NCT02817113	Cisplatin-loaded polymeric micelle, Cetuximab 5-FU	Terminated	Head and Neck Neoplasms	Phase-I	4/30/2021	Strategy change	To evaluate the safety and tolerability profile of NC-6004 in combination with 5- FU plus cetuximab
NCT03531827	Enzalutamide, CRLX101	Terminated	Metastatic Castration Resistant Prostate Cancer, Prostate Neoplasms	Phase -II	7/12/2022	Sever toxicity	To test a new way of treating prostate cancer using CRLX101 plus enzalutamide in people with certain prostate cancer who already had enzalutamide treatment

^*^The investigational armamentarium includes Abemaciclib, Abiraterone, Afatinib (AL), Bevacizumab, bicalutamide, and biospecimen collection procedures; Bortezomib, Cabazitaxel, Cabozantinib, Capecitabine, Carboplatin, Celecoxib, Cobimetinib, Copanlisib, Dabrafenib, Dacomitinib, Darolutamide, Dasatinib, Doxorubicin (AL), Durvalumab, Enasidenib, Entrectinib, Enzalutamide, Erlotinib, Everolimus, Fluorouracil, Idelalisib, Imatinib (AL), Ipilimumab, Lenvatinib, Leucovorin, Lorlatinib, Losartan, Nab-paclitaxel, Neratinib (AL), Nivolumab, Olaparib, Oxaliplatin, Palbociclib, Panobinostat (AL), Pembrolizumab (AL), Pertuzumab, Ponatinib (OTHER), Quality-of-life assessments; Regorafenib, Ruxolitinib, Sirolimus, Sorafenib, Sunitinib, Trametinib (AL), Trastuzumab Emtansine, Tretinoin, Vemurafenib, Venetoclax

### Delivery challenges in the TME

While gene therapy offers powerful modes of action against cancer, the delivery of nucleic acids or genome editors to tumors in vivo remains a central challenge. Systemic administration faces numerous obstacles: ribonucleases and deoxyribonucleases rapidly degrade unprotected RNA/DNA, large complexes such as protein‒nucleic acid assemblies often poorly extravasate into tumor tissue, and immunogenic motifs (e.g., CpG in DNA or long dsRNA structures) can trigger immune activation. The tumor microenvironment (TME), characterized by high interstitial pressure and a dense extracellular matrix (ECM), further hinders the penetration of gene therapy vectors, creating an even greater barrier than small-molecule drugs do [217].

Viral vectors such as adenovirus or adeno-associated virus (AAV) can efficiently transduce dividing and nondividing cells; however, their biodistribution is typically confined to perivascular regions, and they often induce humoral and cellular immune responses, especially with adenoviruses used in both oncolytic and gene therapy trials. Moreover, repeated administration of viral vectors is problematic because of the formation of neutralizing antibodies, diminishing the efficacy of subsequent doses. Nonviral vectors, most notably lipid nanoparticles (LNPs) akin to those used in mRNA vaccines, are gaining prominence for in vivo delivery of siRNA (as exemplified by patisiran) and mRNA therapeutics (Fig. 3). To enhance targeting, LNPs can be functionalized with ligands such as peptides that bind the transferrin receptor or antibody fragments against tumor markers such as EGFR. Despite such targeting, nanoparticle penetration is usually limited to a few cell layers from blood vessels; thus, adjunct strategies such as cancer-associated fibroblast (CAF) depletion or vessel normalization (e.g., via judicious angiogenesis inhibitor use) can augment intertumoral distribution [215, 218, 219]. Another notable challenge is the heterogeneity of target expression within tumors, as not all cancer cells display the intended marker, potentially leaving resistant clones untreated. This limitation might be addressed by combining targeted delivery with enhanced permeability and retention (EPR)-based delivery or by employing carriers with multiple targeting ligands. Furthermore, the acidic and hypoxic microenvironment can impede delivery; for example, in the case of AAV gene vectors, hypoxia may downregulate expression from conventional promoters, although this can be circumvented by using hypoxia-responsive vectors [220, 221]. The integration of nanotechnology into gene therapy, so-called “nanoenabled gene therapy,” is rapidly advancing the field. LNPs excel in siRNA or microRNA delivery, polymeric or gold nanoparticles can carry CRISPR-Cas9 components, and viral vectors may be formulated in hydrogels or delivered via locoregional approaches for enhanced local effects. Overcoming delivery barriers often requires a multifaceted approach: strategies such as ultrasound or electrical fields (electroporation) to transiently permeabilize cell membranes or tumor-penetrating peptides such as iRGD, which promote vascular and stromal permeability, are under active investigation [31]. Despite these formidable challenges, gene therapy for overcoming chemoresistance in cancer is making tangible progress. Notable examples include a phase 1 trial of siG12DLODER, a local polymer system that releases KRAS G12D siRNA for pancreatic cancer, aimed at silencing mutant KRAS and enhancing the response to chemotherapy, and investigations of CRISPR-edited T cells engineered to recognize KRAS G12V in solid tumors [222–224]. In another study, researchers generated CAR-T cells to increase antitumor efficacy, where a CRISPR–Cas9–based strategy was used to disrupt the PD1 gene. These CAR-T cells achieved durable complete remission with high safety in xenograft models and in a phase I trial (NCT04213469), even at low infusion doses [225]. As these technologies mature, it is conceivable that future cancer treatments will include a one-time gene therapy injection designed to eliminate resistance gene subsets or persistently express therapeutic shRNAs, thereby supporting future chemotherapy regimens.

#### Integrated therapeutic models

Given the complexity of chemoresistance, a consensus is emerging that combination strategies are needed to attack cancer on multiple fronts simultaneously. Integrated therapeutic models combine signaling pathway inhibition, epigenetic therapy, and advanced delivery systems (such as nanocarriers) to achieve synergistic effects. This section discusses how dual targeting of signaling and epigenetic axes can enhance treatment efficacy, the development of nanoenabled gene therapy platforms, and several translational examples, including clinical trials (summarized in Table 2), that illustrate these principles. The goal is a multipronged approach using a nanoparticle to codeliver a conventional chemotherapeutic along with an epigenetic drug or a siRNA, thereby modulating the tumor’s biology in a concerted manner.

**Table 2:** Completed clinical trials of nanocomposite-based therapies in chemoresistant cancers. (Includes trials using nanoparticle drug delivery systems with full or partial trial completion status)

NCT Number	Nanocomposite	Study Status	Target tumor	Phase	Last Update Posted
NCT00466960	Paclitaxel albumin-stabilized nanoparticle formulation	Completed	Advanced Epithelial Cancers of the Ovary, Fallopian Tube, and Peritoneum	Phase-II	8/28/2017
NCT03907475	Gemcitabine Hydrochloride, Nab-paclitaxel, Paclitaxel, Pegylated Liposomal Doxorubicin Hydrochloride	Recruiting	Locally Advanced Malignant Solid Neoplasm, Metastatic Malignant Solid Neoplasm	Phase-II	4/9/2025
NCT00499252	Paclitaxel Albumin-Stabilized Nanoparticle Formulation	Completed	Advanced High-Grade Serous Carcinoma of Tubo-Ovarian/Peritoneal Origin	Phase-II	1/11/2018
NCT04734262	Sitravatinib, Tislelizumab, Nab-paclitaxel	Active	Metastatic Breast Cancer	Phase-II	11/18/2023
NCT05832892	Surufatinib, KN046, gemcitabine, Nab paclitaxel	Unknown	Unresectable Locally Advanced or Metastatic Pancreatic Cancer	Phase-I,phase-II	6/14/2024
NCT05422794	BET Bromodomain Inhibitor ZEN-3694, Nab-paclitaxel, Pembrolizumab	Recruiting	Advanced Breast Cancer	Phase-II	7/24/2025
NCT01336062	Nanoparticle Albumin-Bound Paclitaxel	Completed	Gastric Adenocarcinoma	Phase-I,phase-II	5/19/2015
NCT00046514	ABI-007 (A Cremophor-Free, Protein Stabilized, Nanoparticle Paclitaxel)	Completed	Breast Neoplasms, Metastases, Neoplasm	Phase-II	12/15/2016
NCT02631733	Ferumoxytol, Irinotecan Sucrosofate, Veliparib	Completed	Malignant Solid Neoplasm	Phase-I	2/10/2025
NCT02020707	Bevacizumab, Nab-paclitaxel	Completed	Advanced or Refractory Solid Malignancies	Phase-I	7/26/2024
NCT01812746	BIND-014 (Docetaxel Nanoparticles for Injectable Suspension)	Completed	Prostate Cancer	Phase-II	4/15/2016
NCT01652079	CRLX101(a formulation of camptothecin and a large molecule (nanoparticle))	Completed	Ovarian Cancer, Fallopian Tube Cancer, Primary Peritoneal Cancer	Phase-II	7/2/2018
NCT03355261	Carbon nanoparticles (CNs)	Completed	Breast Cancer	Na	2/20/2020
NCT03678883	9-ING-41, Gemcitabine Doxorubicin., Lomustine, Carboplatin., Nab paclitaxel., Paclitaxel., Gemcitabine Irinotecan	Active	Advanced, Metastatic, or Refractory Malignancies	Phase-II	11/7/2024
NCT04669002	EP0057, Olaparib tablets	Completed	Ovarian Cancer	Phase-II	8/2/2023
NCT02369198	TargomiRs (minicells containing a miR-16 microRNA)	Completed	Malignant Pleural Mesothelioma, Small Cell Lung Cancer	Phase-II	4/7/2017
NCT02769962	CRLX101 consists of a sugar molecule cyclodextrin linked to a chemotherapy drug (camptothecin)	Completed	Urothelial Cancer, Small Cell Lung Cancer, Prostate Cancer	Phase-I,phase-II	7/28/2025
NCT03742713	CPC634 (CriPec® docetaxel)	Completed	Ovarian Cancer	Phase-II	12/28/2020
NCT02740985	AZD4635, Durvalumab, Abiraterone Acetate, Enzalutamide, Oleclumab, Docetaxel	Completed	Advanced Solid Malignancies	Phase-I	5/26/2023
NCT01702129	Anti-EGFR immunoliposomes loaded with doxorubicin	Completed	Solid Tumors	Phase-I	9/9/2014
NCT05864534	Balstilimab, Botensilimab, Liposomal Doxorubicin	Recruiting	Glioblastoma	Phase-II	4/29/2025
NCT02507583	IGF-1R/AS ODN; Surgery with tissue harvest and implantation 20 diffusion chambers in the rectus sheath with IGF-1R/AS ODN within 24 h of craniotomy, implanted for 48 h	Completed	Malignant Glioma,Neoplasms	Phase-I	5/7/2025
NCT03608631	Mesenchymal Stromal Cells-derived Exosomes with KRAS G12D siRNA	Active	KRAS G12D-Mutant Metastatic Pancreatic Adenocarcinoma	Phase-I	4/23/2025
NCT01550523	IGF-1R/AS ODN, autologous tumor cells exosomes	Completed	Malignant Glioma of Brain	Phase-I	5/2/2025
NCT05666700	Cilta-cel, & Super Paramagnetic Iron Oxide Nanoparticle (64Cu SPION)	Recruiting	Extramedullary Myeloma	Phase-I	4/11/2024
NCT05161507	Superparamagnetic Iron Oxide Nanoparticles	Completed	Node-Positive Breast Cancer	Observational	1/26/2024
NCT05625698	Superparamagnetic Iron Oxide Nanoparticles	Completed	Node-Positive Breast Cancer	Interventional	1/26/2024
NCT05985551	Superparamagnetic Iron Oxide Nanoparticles	Active	Breast Cancer	Observational	5/3/2024
NCT06572475	Superparamagnetic Iron Oxide Nanoparticles	Recruiting	Vestibular Schwannoma, Glioma, Astrocytic	Interventional	8/27/2024
NCT05703971	Tumor Suppressor Gene 2 (TUSC2), Lipid Nanoparticle (LNP),	Recruiting	Small Cell Lung Cancer Extensive Stage	Phase-I, phase-II	2/25/2025
NCT01437007	TKM-080301	Completed	Colorectal, Pancreas, Gastric, Breast Ovarian Cancer with Hepatic Metastases	Phase-I	8/3/2018
NCT04486833	Quaratusugene ozeplasmid, osimertinib, Platinum-Based Chemotherapy	Recruiting	Carcinoma, Non-Small Cell Lung	Phase-I,phase -II	3/25/2025
NCT04950166	Pegsitacianine	Completed	Peritoneal Carcinomatosis	Phase-II	11/8/2024
NCT02106598	Fluorescent cRGDY-PEG-Cy5.5-C dots	Active	Head and Neck Melanoma	Phase-I, phase-II	5/2/2025

#### Dual targeting of signaling and epigenetic axes

One rational combination is to pair inhibitors of key oncogenic signaling pathways with epigenetic modulators to prevent or reverse the adaptive responses that lead to resistance. For example, in BRAF-mutant melanoma, the initial response to BRAF inhibitors is high, but resistance often develops via reactivation of MAPK or activation of parallel pathways such as the PI3K/AKT pathway [226]. Tumors also show transcriptional/epigenetic changes during acquired resistance. Preclinical studies and early trials have explored the addition of an HDAC inhibitor or DNA methylation inhibitor to targeted therapy to keep the tumor “differentiated” and less able to adapt. One study showed that the HDAC inhibitor panobinostat could prevent the emergence of resistance to BRAF/MEK inhibitors in melanoma by blocking the drug-induced upregulation of MITF and other survival factors [227].

Another strategy under investigation is the combination of EGFR tyrosine kinase inhibitors (TKIs) with epigenetic therapies. Resistance to EGFR inhibitors in lung cancer frequently involves epithelial-to-mesenchymal transition (EMT) or lineage plasticity, processes often driven by epigenetic silencing of key regulatory genes. A phase I trial tested the DNA methyltransferase inhibitor azacitidine in combination with the EGFR TKI erlotinib and reported that the regimen was safe and tolerable, establishing recommended doses for future studies, although clear evidence of restored TKI sensitivity was not observed in that early study [228]. The rationale is that azacitidine can demethylate and turn back on genes such as *mir200,* which suppresses EMT, or *NKX21,* a lineage transcription factor, thereby reversing EMT and restoring dependency on EGFR signaling, making cancer cells vulnerable again to EGFR blockade [229]. Although small, that trial revealed partial responses in patients who had progressed on erlotinib, indicating that epigenetic therapy helped overcome resistance. The combination of chemotherapy plus an epigenetic drug is another dual approach. HDAC inhibitors can acetylate histones and nonhistone proteins (such as p53) to enhance the DNA damage response. In a phase I/II study in advanced lung cancer, adding an HDAC inhibitor to pemetrexed chemotherapy improved progression-free survival to some extent, particularly in patients who had low baseline expression of a certain gene, suggesting that epigenetic modulation is beneficial. Similarly, histone deacetylase inhibitors have been combined with platinum-based chemotherapies in ovarian and other cancers; these agents seem to resensitize platinum-resistant tumors by re-expressing genes involved in DNA mismatch repair and apoptosis [75]. They discussed the use of hydralazine plus valproate to resensitize a variety of refractory tumors and reported 80% disease control in heavily pretreated patients. The chemotherapeutics hit the DNA, and the epigenetic drugs likely made the cancer DNA more accessible and the cells less able to repair or bypass the damage, which strongly supports dual targeting [75]. Another promising dual-targeting therapeutic strategy in cancer treatment involves simultaneous targeting of a survival pathway, such as PI3K, plus epigenetic regulators, such as histone deacetylases (HDACs) [230]. The PI3K/AKT signaling axis is frequently upregulated in malignancies, leading to downstream effects such as phosphorylation and inactivation of glycogen synthase kinase 3β (GSK3β) [231]. This process can influence chromatin structure by stabilizing oncogenic transcription factors, ultimately contributing to tumor cell survival and proliferation. Recent studies have demonstrated that combining a PI3K inhibitor with an HDAC inhibitor results in synergistic antitumor activity in lymphoma and various solid tumors [232]. Mechanistically, HDAC inhibition can reduce the expression of compensatory survival proteins, thereby sensitizing cancer cells to PI3K inhibitor-induced apoptosis, an effect that can be further amplified by an HDAC inhibitor. Several novel dual PI3K/HDAC inhibitors, such as CUDC-907, have entered clinical or preclinical evaluation, showing enhanced efficacy over individual agents and providing durable responses in certain cancer subtypes [233, 234]. This combination approach not only increases direct cytotoxicity but also addresses resistance mechanisms and tumor heterogeneity, supporting its potential application in relapsed or refractory cancers and as part of rational combination therapies in the broader oncology landscape.

#### Nanoenabled gene therapy systems

Nanocarriers are increasingly being used as platforms to deliver combination therapies in one package, for instance, delivering a chemotherapeutic drug along with a siRNA or CRISPR system [35, 37, 235]. We previously discussed codelivery with siRNA in the nanotech section; here, we focus on integration with gene editing tools. One innovative concept is an “all-in-one” nanoparticle capable of carrying a Cas9 plasmid and a chemotherapy prodrug. For example, Pan et al. introduced a self-amplifying, logic-gated nanoplatform for precise tumor-targeted cancer therapy by combining gene editing and metabolic disruption. The system uses a hypoxia-sensitive covalent organic framework (COF) coated with a-ZIF-8 nanoparticles, which carry glucose oxidase (GOx), a CRISPR gene-editing system, and DNAzymes that degrade catalase mRNA to disrupt tumor antioxidant defenses. Upon entering the acidic and hypoxic tumor microenvironment, the nanoparticles degrade, activating GOx to increase the intracellular acidity and hypoxia, which further accelerates nanoparticle breakdown and the release of CRISPR plasmids. This cascade depletes glucose and oxygen, triggering targeted gene editing and synergistic cancer cell starvation through H2O2 generation. Overall, this biocomputing-inspired strategy enhances CRISPR delivery and offers highly precise, multimodal cancer therapy with significant potential for improved treatment outcomes [196].

Another notable example involves codelivery of CRISPR and paclitaxel via acidity-responsive nanoparticles. Another study showed that encapsulated Cas9 targeted CSF1 to deplete tumor-associated macrophages along with paclitaxel; in the acidic tumor microenvironment, the nanoparticles released both CRISPR components to edit immunosuppressive macrophages and paclitaxel to kill tumor cells. This combined release converts macrophage-rich, resistant tumors into those responsive to therapy [236]. These integrated nanosystems underscore how multiple layers of immune and intrinsic tumor cells can be approached with one therapeutic. These integrated strategies yield encouraging results. The epigenetic and chemical approaches demonstrated real patient benefit in a difficult population [75]. Mouse xenograft models show that nanoparticle-facilitated gene therapy (siRNA) could potentially achieve outcomes beyond what standard therapy alone can achieve in preclinical settings (Fig. 3), though these findings warrant further investigation in clinical contexts. CRISPR-edited cell therapies are moving through early-phase trials with preliminary evidence suggesting acceptable safety profiles. While these findings are encouraging, they remain early-phase results that suggest a potential mechanism for next-generation combination regimens rather than established clinical efficacy and warrants further validation in larger randomized trials. A “universal sensitizing regimen” for solid tumors might consist of a low-dose epigenetic drug to prime the tumor, a nanoparticle delivering both a chemotherapeutic agent and a gene-silencing agent, and an immunotherapy component to eliminate residual disease. Each element targets a distinct facet of resistance, including intrinsic cellular mechanisms, the tumor microenvironment, and adaptive responses.

### Translational & future perspectives

As our understanding of chemoresistance deepens, personalization and combination therapies will be crucial for future cancer treatment. Tumors from different patients, even those of the same histologic subtype, may rely on distinct resistance mechanisms. For example, one tumor might exhibit strong PI3K/AKT-driven resistance, whereas another depends on an EMT program coupled with epigenetic silencing. This paves the way for personalized epigenetic-guided therapies, where molecular profiling of a patient’s tumor identifies key signaling pathways and epigenetic alterations to tailor treatment. For example, tumors with hypermethylation of multiple tumor suppressors may respond well to hypomethylating agents, whereas tumors driven by NF-κB signaling might benefit from a combination of IKK inhibitors and nanoparticles delivering NF-κB-targeting siRNAs. Advanced diagnostic techniques such as RNA sequencing and ATAC-seq, which assess chromatin accessibility, can reveal active gene programs and epigenetically silenced regions from tumor biopsies. Additionally, emerging blood-based biomarkers, including circulating tumor DNA methylation patterns and exosomal miRNA profiles, hold promise for real-time monitoring of epigenetic therapy efficacy [85]. In the future, adaptive clinical trials could enable patients to start with baseline chemotherapy or targeted therapy. On the basis of early molecular feedback, such as persistent methylation of key genes or increased expression of certain cytokines, an adaptive addition of a specific inhibitor or gene therapy could be added. This allows real-time personalized treatment to overcome resistance. One concept is a theragnostic nanoparticle that, after delivering a drug, can report by imaging or a detectable marker, whether the targeted pathway is inhibited or a particular population is killed [114]. For example, a nanoparticle carrying a drug could be tagged with an MRI contrast agent and a moiety that binds to an apoptosis marker; in imaging, one could observe drug release and tumor cell apoptosis in real time, allowing early assessment of the response [237]. If parts of the tumor do not respond to no apoptosis signal, clinicians could intervene sooner with a different strategy instead of waiting for tumor growth on scans. Another futuristic approach is the use of logic-gated circuits in cell therapies, such as CAR-T cells, which are activated only under certain TME conditions and can act as sensors of the microenvironment and deliver biologics accordingly. For example, a synthetic biological circuit in a cell could detect high levels of TGFβ, which is a signature of the immunosuppressive TME, and in response, secrete an epigenetic drug locally or a cytokine to modulate the environment. Such precision might mitigate side effects and maximize the impact on resistant niches.

Based on gene therapy, base editors and prime editors (such as NextGen CRISPR tools that can make precise nucleotide changes without double-strand breaks) could correct mutations that cause resistance, such as secondary mutations in a drug target gene. One could deliver a base editor to convert a resistance-conferring mutation back to wild type, restoring drug sensitivity, although this is very sci-fi at present and would require extremely efficient tumor editing.

### Overcoming challenges

Despite progress, several challenges remain for future work. One is the potential toxicity and interactions of combining multiple powerful agents. Epigenetic drugs can have systemic side effects, such as DNA hypomethylation, which affects blood cell counts, and HDAC inhibitors can cause fatigue and thrombocytopenia. Finding the right dosing schedule may lead to short-term “pulses” of epigenetic drugs to prime tumors, which can then be stopped when administering chemotherapy is important to minimize added toxicity. Nanocarriers can trigger immune reactions, and the addition of targeting ligands or multiple payloads increases complexity. Careful testing in preclinical models that mimic the human tumor microenvironment, such as patient-derived xenografts or organoids with stromal cells, is needed to ensure safety and benefit. Another challenge is tumor heterogeneity and evolution. A drug cocktail might eliminate 99% of cells, but a small resistant population not covered by the cocktail will eventually grow back. This calls for broad-spectrum approaches [70]. Epigenetic drugs are interesting because they act in a genome-wide manner and can affect many pathways simultaneously, but this also means unpredictability. Artificial intelligence and network modeling may help predict which combination of pathways plus epigenetic targets covers the most ground for a given tumor profile.

### Clinical trial data

To date, combination approaches are still emerging, but early-phase trials have shown promising improvements. The key to designing trials with proper biomarkers, such as correlating demethylation with EGFR TKI resensitization in lung cancer, is critical for identifying responders. There are also regulatory and logistical challenges in developing therapies that involve multiple components. Tables 3 and 4 are presented as illustrative of the landscape and challenges, not as comprehensive evidence of clinical translation. Phase II/III demonstrated efficacy is explicitly noted where applicable; all other entries represent early-phase or investigational findings that warrant further validation. The key to designing trials with proper biomarkers, such as correlating demethylation with EGFR TKI resensitization in lung cancer, is critical for identifying responders. There are also regulatory and logistical challenges in developing therapies that involve multiple components (e.g., a drug plus a device or a cell therapy plus a nanoparticle), but regulatory pathways (FDAs) are adapting new guidance for combination products and individualized gene therapies.

**Table 3:** Clinical Trials of Gene-Targeting and Combination Immunotherapies in Patients With Chemoresistance

Trial-ID	Interventions	Study Status	Target Tumor	Phases	Last Posted
NCT00470704	Lapatinib, Herceptin	Completed	Breast Cancer	Phase-II	12/19/2024
NCT03597685	Biopsies sampling	Completed	Lymphoma Diffuse Large B-cell	Observational	4/18/2019
NCT05564650	Navitoclax,Venetoclax, Decitabine	Active	Myelodysplastic Syndrome (MDS)	Phase-I, phase-II	2/11/2025
NCT02650986	Cyclophosphamide, Decitabine, TGFbDNRII-transduced Autologous Tumor Infiltrating Lymphocytes	Active	Advanced or Refractory Solid Malignancies	Phase-I, phase-II	6/17/2025
NCT06501391	PD-L1/PD-1 inhibitor and chemotherapy, SRT or WBRT	Recruiting	Non-Small Cell Lung Cancer with Brain Metastases	Phase-II	8/28/2024
NCT00557193	Asparaginase, Cyclophosphamide, Cytarabine, Daunorubicin Hydrochloride, Dexamethasone, Etoposide, Filgrastim, Lestaurtinib, Leucovorin Calcium, Mercaptopurine, Methotrexate, Methylprednisolone, Pegaspargase, Prednisone, Therapeutic Hydrocortisone, Vincristine Sulfate	Completed	Acute Lymphoblastic Leukemia (ALL)	Phase-III	7/15/2024
NCT06219941	AZD0901,5-Fluorouracil, Leucovorin, l-leucovorin, Irinotecan, Nanoliposomal Irinotecan, Gemcitabine	Recruiting	Upper Gastrointestinal (GI) Cancers	Phase-II	7/7/2025
NCT00879385	Decitabine, Panitumumab	Completed	Colorectal Cancer	Phase-I	10/31/2014
NCT06233526	Chemotherapy regiments based on the transcriptomic profile and in vitro drug sensitivity test	Recruiting	Acute Myeloid Leukemia in Children	Interventional	7/3/2024
NCT06236139	Anti-STEAP1 CAR T cells, Cyclophosphamide, Enzalutamide, Fludarabine	Recruiting	Metastatic Prostate Cancer	Phase-I, phase-II	3/30/2025
NCT04015739	Bevacizumab, Olaparib and Durvalumab (MEDI 4736) combination	Unknown	Recurrent Epithelial Ovarian Cancer	Phase-II	8/17/2022
NCT05334277	Furmonertinib, Furmonertinib, Furmonertinib/Pemetrexed/Carboplatin, Furmonertinib/Pemetrexed/Carboplatin/Bevacizumab	Recruiting	Non-small Cell Lung Cancer	Phase-II	8/31/2022
NCT04488783	Zinc and ascorbate in Diet	Unknown	Newly Diagnosed Glioblastoma, Status Post-Resection	Na	7/28/2020
NCT04253145	PM 01183, Atezolizumab	Recruiting	Small Cell Lung Cancer (SCLC)	Phase-I, phase-II	11/18/2023
NCT07058519	Osimertinib-based adaptive treatment, Osimertinib 80 MG	Not yet recruiting	Locally Advanced or Metastatic EGFRm Non-small Cell Lung Cancer (NSCLC)	Phase-II	7/10/2025
NCT05633602	Chemotherapy, Pembrolizumab, Ramucirumab	Active	Advanced or Metastatic Non-Small Cell Lung Cancer (NSCLC)	Phase-III	5/28/2025
NCT05702619	Pimonidazole Hydrochloride	Unknown	Prostate Cancer and Hypoxia	Observational	1/27/2023
NCT02098954	Gemcitabine platinum combined with erlotinib	Recruiting	EGFR-Mutant Non-Small Cell Lung Carcinoma	Phase-II	12/27/2023
NCT03791151	Cisplatin	Unknown	Non-Small Cell Lung Cancer	Observational	1/2/2019
NCT04345913	Copanlisib Hydrochloride, Eribulin Mesylate	Active	Advanced Breast Cancer	Phase-I, phase-II	7/28/2025
NCT05312398	Cetuximab, FOLFIRI, FOLFOX regimen, Irinotecan	Active	Metastatic Colorectal Adenocarcinoma	Phase-II	5/25/2025
NCT06228404	Enhanced autologous PSMA-CAR T	Recruiting	Castration-resistant Prostate Cancer	Phase-I	4/18/2024
NCT04691284	Blood, urine, and stool sampling	Recruiting	Hematologic Neoplasms	Interventional	5/29/2025
NCT06906978	Blood sampling		Acute Myeloid Leukemia	Interventional	4/10/2025
NCT06439225	Docetaxel, Carboplatin	Recruiting	Metastatic Prostate Cancer	Phase-III	6/11/2025
NCT05052957	P140K-MGMT, O6-benzylguanine, Photon Based Radiotherapy, temozolomide, Filgrastim, carmustine	Recruiting	Glioblastoma	Phase-II	3/11/2025
NCT01942837	Enzalutamide	Completed	Metastatic Castration-resistant Prostate Cancer	Phase-II	10/24/2022
NCT06863818	Blood and Biopsies sampling	Active	Non-Small Cell Lung Cancer	Observational	3/7/2025
NCT03854032	Nivolumab, IDO1 Inhibitor BMS-986205	Active	Head and Neck Squamous Cell Carcinoma (HNSCC)	Phase-II	6/6/2025
NCT05563766	Itraconazole	Recruiting	Esophageal Adenocarcinoma,Esophageal Squamous Cell Carcinoma,Gastroesophageal Junction Carcinoma	Phase-II	11/21/2024
NCT02977468	Pembrolizumab, Intraoperative radiation therapy (IORT)	Recruiting	Triple Negative Breast Cancer	Phase-II	6/20/2024
NCT03410030	Ascorbic Acid, Paclitaxel protein-bound, Cisplatin, Gemcitabine	Completed	Pancreatic Cancer, Pancreatic Adenocarcinoma Resectable,Pancreatic Ductal Adenocarcinoma, Pancreas Metastases	Phase-I, phase-II	4/18/2023
NCT06894225	ACT001	Not yet recruiting	Recurrent Glioblastoma	Phase-II	4/6/2025

**Table 4:** Terminated or Withdrawn Gene-Based and Immunotherapy Trials Related to Chemoresistance

Trial-ID	Interventions	Study Status	Target Tumor	Phases	Last Update Posted	Reason	Expert Insight (Critical Perspective)
NCT00272870	Benzylguanine, Temozolomide, Hematopoietic stem cells transduced MGMTP140K gene	Terminated	High-grade glioma	Phase-I	8/29/2012	Slow patient enrollment and drug unavailability	This trial sensitizes tumors to Temozolomide using O6-Benzylguanine, while protecting the patient's blood stem cells from toxicity with MGMTP140K gene therapy
NCT02793765	Docetaxel, Sipuleucel-T	Withdrawn	Prostate cancer	Phase-II	7/30/2017	Due to corporate acquisition (New company stopped funding)	The combination of docetaxel followed by Provenge (sipuleucel-T)
NCT02949843	Nivolumab, Pembrolizumab, Tyrosine Kinase Inhibitor	Terminated	Advanced egfr-mutant non-small cell lung cancer (nsclc)	Phase-II	6/28/2024	By the IRB due to slow enrollment	Objective Response Rate (ORR) of subsequent-line immunotherapy in patients with incurable, PD-L1-high non-small cell lung cancer harboring an activating driver mutation (EGFR, ALK, BRAF, etc.) whose disease is refractory to prior targeted therapy
NCT02392377	Carboplatin and paclitaxel or oxaliplatin, leucovorin calcium, and fluorouracil, Radiation Therapy	Terminated	Locally advanced esophageal adenocarcinoma	Phase-II	2/13/2018	Sow patient enrollment	To correlate pretreatment gene and miRNA expression with chemotherapy response
NCT01548573	Dexamethasone, autologous stem cell transplant, Cisplatin, Doxorubicin, Cyclophosphamide, Etoposide, Bortezomib, Thalidomide, Melphalan	Terminated	Multiple myeloma	Phase-II	6/14/2017	Met study stopping rules	To evaluated gene expression profiles from patient biospecimens to better understand drug resistance and identify novel therapeutic targets
NCT00098371	Alvocidib	Terminated	Advanced or high-risk b-cell malignancies	Phase-II	10/24/2016	Cancer Therapy Evaluation Program (CTEP) initiated	To assess safety and toxicity profile including biomarkers to understand the drug's mechanism of action and the development of resistance

One promising frontier is immune epigenetic therapy, which combines epigenetic drugs with immunotherapy. Preclinical and early clinical evidence suggests epigenetic agents may upregulate MHC molecules and tumor antigens, converting "cold" tumors into immunogenic ones.

One promising frontier is immune epigenetic therapy, which combines epigenetic drugs with immunotherapy. Preclinical and early clinical evidence suggests epigenetic agents may upregulate MHC molecules and tumor antigens, converting "cold" tumors into immunogenic ones [99]. Clinical trials, such as those combining the DNMT inhibitor guadecitabine with PD-1 checkpoint blockade in lung cancer, have indicated improved response rates in previously resistant patients. In essence, overcoming chemoresistance might also address monoresistance; these two mechanisms are intertwined if chemotherapy fails, and immunotherapy alone often does not work either because the tumor likely has multiple protective mechanisms [238]. Chemoresistance and monoresistance often overlap, suggesting that future regimens could involve triple combinations of targeted therapy, epigenetic drugs, and immunotherapy or chemotherapy plus epigenetic plus immunotherapy, potentially orchestrated via nanotechnology. Finally, prevention of resistance is as important as treatment. Neoadjuvant or first-line therapies of the future might include an epigenetic modulator from the beginning to prevent the tumor from ever “learning” resistance. For example, administering a low-dose HDAC inhibitor alongside first-line chemotherapy in triple-negative breast cancer could hypothetically keep the tumor cells in a state that is easier for the chemo to kill and less likely to spawn resistant clones. Clinical trials are needed to confirm that this yields better long-term outcomes. In conclusion, the landscape of overcoming chemoresistance is moving toward multimodal, personalized therapy. By transforming the tumor microenvironment, which makes it more perfusable, immunoactive, and less protective and simultaneously attacks the internal resistance circuits (signaling and epigenetic) of cancer cells, these advanced strategies offer hope for more durable responses. The integration of nanotechnology and gene therapy adds precision and firepower to conventional treatments, marking a paradigm shift in cancer therapy. While challenges remain, the convergence of these fields (cancer biology, nanomedicine, epigenetics, and immunotherapy) sets the stage for therapies that adapt to and outsmart tumor evolution. The ultimate goal is to render even aggressive, previously drug-resistant cancers manageable or curable through smart combination therapies that leave no escape route for cancer.

#### Nanocomposite-based clinical trials targeting chemoresistance

Recent clinical trials underscore a growing emphasis on harnessing nanocomposite platforms to overcome chemoresistance in a wide array of malignancies, as discussed in Table 3. Completed clinical trials in the nanomedicine field have revealed substantial progress in the use of nanoparticle-based drug delivery systems to address chemoresistance. These studies predominantly reached phase I or II and included diverse malignancies, including advanced ovarian, breast, gastric, pancreatic, prostate, and glioma cancers. Key nanocomposite formulations, such as paclitaxel albumin-stabilized nanoparticles, CRLX101, and nanoparticle albumin-bound paclitaxel, have been widely tested and have demonstrated versatility and promising efficacy across tumor types. Innovative therapeutic agents such as TargomiRs, which deliver microRNAs, and exosomes loaded with siRNA highlight the integration of molecular targeting within nanomedicine platforms [239, 240]. This body of research underscores the potential of nanotechnology to increase drug delivery, modulate resistance pathways, and improve therapeutic outcomes. Despite these advances, several nanocomposite trials have been terminated, exposing the ongoing challenges discussed in Table 4. The reasons for termination included poor patient accrual, toxicity concerns, insufficient efficacy, and changes in strategic development priorities. Notably, terminated studies involve investigational armamentariums composed of multiple agents, dual Bcl-2/Bcl-xL inhibitors, and combination therapies with cisplatin-loaded micelles. Some trials, such as those investigating TUSC2 tumor suppressor gene nanoparticles, faced slow enrollment compounded by competition from other studies. These obstacles reflect the complexity of trial execution and the critical need for improved patient recruitment, safety monitoring, and adaptive clinical strategies to translate nanomedicine innovations effectively.

#### Gene-based and combination immunotherapy trials in chemoresistance

Parallel efforts in gene-targeting and immunotherapy have yielded successfully completed trials that employ molecularly tailored strategies against chemoresistant cancers, as discussed in Table 1. Examples include dual HER2 blockade with lapatinib and trastuzumab in patients with breast cancer, multiagent chemotherapy regimens in patients with acute lymphoblastic leukemia, and CAR-T-cell therapies combined with chemotherapeutic agents in patients with metastatic prostate cancer. Many studies have incorporated detailed molecular profiling to customize treatment and monitor resistance mechanisms. These completed trials highlight the feasibility and clinical potential of integrating gene-targeted approaches and immuno-oncology agents to overcome resistance and improve patient outcomes. Several gene-focused trials were discontinued owing to slow patient enrollment, drug availability issues, funding interruptions, and safety concerns (discussed in Table 3). For example, trials evaluating gene therapy for high-grade glioma, immune checkpoint inhibitors for EGFR-mutant lung cancer, and combination immunotherapy for prostate cancer were terminated or withdrawn owing to these challenges. Institutional review board decisions also played a role in halting some studies, emphasizing the stringent oversight required in early-phase gene therapy and immunomodulation trials. These terminations highlight the inherent complexities of translating cutting-edge gene therapies into clinical practice and underscore the importance of adaptive trial designs, robust recruitment strategies, and sustained funding support.

Together, these clinical trial landscapes reveal a dynamic but challenging path toward overcoming chemoresistance through nanomedicine and gene-based therapies. Completed studies showcase innovative therapeutic approaches with encouraging results, whereas terminated trials provide critical insights to optimize future development. Continued efforts in refining trial designs, enhancing patient engagement, and understanding resistance biology are essential for advancing these promising modalities into effective clinical treatments.

### FDA-withdrawn therapeutic agents

Several targeted and chemotherapeutic agents that once received FDA approval for the treatment of various malignancies have subsequently been withdrawn due to issues including lack of confirmed benefit, toxicity, or failure to meet post marketing requirements (Table 5) [241]. Recently, withdrawn agents included Exkivity (mobocertinib) for EGFR exon 20 insertion-mutant non-small cell lung cancer, Truseltiq (infigratinib) for cholangiocarcinoma, and Pepaxto (melphalan flufenamide) for relapsed or refractory multiple myeloma, all of which were approved under the FDA’s accelerated program but were later removed after confirmatory studies failed to demonstrate adequate clinical benefit. Other high-profile withdrawals include immune checkpoint inhibitors such as Keytruda (pembrolizumab), Opdivo (nivolumab), and Tecentriq (atezolizumab) for certain indications, as well as targeted therapies such as Ukoniq (umbralisib) and Gavreto (pralsetinib). These withdrawals reflect the dynamic regulatory environment and underscore the importance of rigorous postapproval evaluation in oncology. Importantly, several of these drugs specifically target mechanisms associated with resistance, highlighting the complexity of overcoming chemoresistance in real-world settings. These regulatory actions serve to protect patients but also reveal the ongoing challenges in translating initial efficacy signals into durable, meaningful improvements in survival and quality of life for cancer patients [241, 242]. This approach also demonstrates the value of adaptive drug development and the necessity for robust postapproval clinical trials to verify real-world benefits in patients with chemoresistant and otherwise difficult-to-treat cancers. Recent developments in oncology drugs, including more precise kinase inhibitors, bispecific antibodies, and advanced immunotherapies such as CAR-T cells and immune checkpoint inhibitors that more effectively target molecular drivers of resistance, have resulted in new approaches [243]. However, the elevated pace of withdrawal alongside new approvals underscores the need for rigorous postmarketing surveillance and an adaptable, mechanism-driven approach to overcome cancer chemoresistance.

## Concluding remarks, limitations and future directions

Despite the promise of TME-targeted nanotherapeutic and gene therapy strategies, several critical limitations must be acknowledged. The enhanced permeability and retention (EPR) effect varies substantially across tumor types, anatomical locations, and individual patients [221]. Not all solid tumors exhibit sufficient vascular permeability or impaired lymphatic drainage to enable effective nanoparticle accumulation [6]. This heterogeneity limits the universal applicability of passive targeting strategies [6]. Even when nanoparticles reach the tumor vasculature, dense stromal matrices, elevated interstitial fluid pressure, and heterogeneous perfusion impede deep penetration into tumor tissue. Dense extracellular matrix, elevated interstitial fluid pressure, and stromal cell populations create physical and biochemical barriers that limit nanoparticle penetration into tumor parenchyma, particularly in desmoplastic tumors such as pancreatic adenocarcinoma. This results in uneven drug distribution and incomplete coverage of resistant cell populations. Viral and non-viral gene delivery systems face challenges including low transfection efficiency in vivo, immune clearance, and difficulty targeting specific cell populations within the TME [41]. While gene therapy holds immense promise, in vivo transfection efficiency remains low, particularly for non-viral vectors. Immune clearance of both viral and non-viral vectors, off-target CRISPR-Cas9 editing, and transient expression of therapeutic genes represent significant technical hurdles [34, 244]. CRISPR-based approaches require precise delivery to achieve therapeutic gene editing without off-target effects. Both nanocarriers and gene therapy vectors can trigger immune responses, leading to accelerated clearance, inflammatory toxicity, and reduced therapeutic efficacy. Both nanocarriers (particularly those containing cationic lipids or polymers) and gene therapy vectors (especially viral vectors) can trigger innate and adaptive immune responses, leading to rapid clearance, reduced efficacy, and potential toxicity [34, 244]. Identifying robust, clinically validated biomarkers to predict which patients will respond to TME-targeted or epigenetically focused therapies remains a major challenge [244]. Without reliable stratification tools, clinical trial outcomes will remain variable. Despite the central role of tumor-acquired resistance in undermining targeted therapies, regulatory decisions and drug withdrawals seldom cite resistance as the official cause. For example, the development of rociletinib, a third-generation EGFRTKI developed to overcome the T790M mutation in NSCLC, was halted by Clovis in 2016 after disappointing phase II/III data and the emergence of the competitor osimertinib, despite its early promise in resistant tumors [245, 246]. However, the public rationale focused on the lack of sufficient response rates and regulatory nonapproval, not the mechanistic reality that resistance subclones continue to emerge. Similarly, Iniparib, initially marketed as a PARP inhibitor for triple-negative breast cancer, was discontinued after phase III trials revealed no survival benefit, but retrospective analyses revealed that it failed to engage its target and cross-resistance to platinum therapy, likely underpinning its ineffectiveness, not classical chemoresistance per se. These examples underscore a critical communication gap: while resistance mechanisms such as EGFR mutation resurgence or PARP inhibitor escape pathways are well characterized biologically, clinical trial failures are recorded under broader terms such as “lack of efficacy” or “failure to confirm clinical benefit.” Thus, although drug resistance is a fundamental driver of treatment failure, it remains subsumed under outcome-based classifications, leaving both clinicians and the public unaware of the mechanistic underpinnings behind high-profile pipeline collapses. Complex nanomedicines and gene therapies face stringent regulatory requirements and manufacturing challenges that limit scalability and increase costs. Future research must prioritize: (1) developing predictive biomarkers for patient selection; (2) engineering next generation nanocarriers with improved penetration and targeting; (3) combining multiple therapeutic modalities (chemotherapy + immunotherapy + epigenetic drugs); (4) establishing standardized preclinical models that better recapitulate human TME complexity; and (5) conducting rigorously designed clinical trials with appropriate endpoints.

**Table 5:** FDA-withdrawn therapeutic agents in oncology: withdrawals and rationale

Drug Name	Indication	Approval Date	Withdrawal Date
Exkivity (mobocertinib)	Treatment of adult patients with locally advanced or metastatic non-small cell lung cancer (NSCLC) with EGFR exon 20 insertion mutations, as detected by an FDA-approved test, whose disease has progressed on or after platinum-based chemotherapy	9/15/2021	7/15/2024
Truseltiq (infigratinib)	Treatment of adult patients with previously treated unresectable locally advanced or metastatic cholangiocarcinoma with fibroblast growth factor receptor 2 (FGFR2) gene fusions or other rearrangement as detected by an FDA approved test	5/28/2021	5/16/2024
Trodelvy (sacituzumab govitecan-hziy)	Treatment of adult patients with locally advanced or metastatic urothelial cancer (mUC) who have previously received a platinum-containing chemotherapy and either programmed death receptor-1 (PD-1) or programmed death-ligand 1 (PD-L1) inhibitor	4/13/2021	11/22/2024
Pepaxto (melphalan flufenamide)	In combination with dexamethasone for the treatment of adult patients with relapsed or refractory (R/R) multiple myeloma (MM) who have received at least four prior lines of therapy and whose disease is refractory to at least one proteasome inhibitor, one immunomodulatory agent, and one CD-38 directed monoclonal antibody	2/26/2021	2/23/2024
Ukoniq (umbralisib)	Adult patients with relapsed or refractory marginal zone lymphoma (MZL) who have received at least one prior anti-CD20- based regimen	2/5/2021	5/31/2022
Ukoniq (umbralisib)	Adult patients with relapsed or refractory follicular lymphoma (FL) who have received at least three prior lines of systemic therapy	2/5/2021	5/31/2022
Gavreto (pralsetinib)	Treatment of adult and pediatric patients 12 years of age and older with advanced or metastatic RET-mutant medullary thyroid cancer (MTC) who require systemic therapy	12/1/2020	7/20/2023
Blenrep (belantamab mafodotin-blmf)	Adult patients with relapsed or refractory (R/R) multiple myeloma (MM) who have received at least four prior therapies including an anti-CD38 monoclonal antibody, a proteasome inhibitor, and an immunomodulatory agent	8/5/2020	2/6/2023
Keytruda (pembrolizumab)	Metastatic SCLC with disease progression on or after platinum-based chemotherapy and at least one other prior line of therapy	6/17/2019	3/30/2021
Tecentriq (atezolizumab)	In combination with paclitaxel protein-bound for unresectable locally advanced or metastatic triple-negative breast cancer whose tumors express PD-L1 (PD-L1 stained tumor-infiltrating immune cells of any intensity covering = 1% of the tumor area), as determined by an FDA-approved test	3/8/2019	10/6/2021
Copiktra (duvelisib)	Treatment of adult patients with relapsed or refractory follicular lymphoma (FL) after at least 2 prior systemic therapies	9/24/2018	12/17/2021
Opdivo (nivolumab)	Metastatic SCLC with progression after platinum-based chemotherapy and at least one other line of therapy	8/16/2018	12/29/2020
Keytruda (pembrolizumab)	For patients with recurrent or locally advanced or metastatic gastric or GEJ adenocarcinoma whose tumors express PD-L1 as determined by an FDA-approved test, with disease progression on/after two or more prior lines of therapy including fluoropyrimidine and platinum containing chemotherapy and if appropriate, HER2/NEU targeted therapy	9/22/2017	2/4/2022
Opdivo (nivolumab)	Hepatocellular carcinoma previously treated with sorafenib	9/22/2017	7/23/2021
Aliqopa (copanlisib)	Treatment of adult patients with relapsed follicular lymphoma (FL) who have received at least two prior systemic therapies	9/14/2017	3/18/2024
Imfinzi (durvalumab)	Locally advanced or metastatic urothelial carcinoma that progressed during or following platinum-containing chemotherapy or within 12 months of neoadjuvant or adjuvant treatment with platinum-containing chemotherapy	5/1/2017	2/19/2021
Tecentriq (atezolizumab)	Patients with locally advanced or metastatic urothelial carcinoma (mUC) who are not eligible for cisplatin-containing chemotherapy and whose tumors express PD-L1 as determined by an FDA approved test or who are not eligible for any platinum-containing chemotherapy regardless of PD-L1 status	4/17/2017	12/2/2022
Imbruvica (ibrutinib)	Adult patients with marginal zone lymphoma (MZL) who require systemic therapy and have received at least one prior anti-CD20-based therapy	1/18/2017	5/18/2023
Lartruvo (olaratumab)	In combination with doxorubicin for adults with soft tissue sarcoma with a histologic subtype for which an anthracycline-containing regimen is appropriate and which is not amenable to curative treatment with radiotherapy or surgery	10/19/2016	2/25/2020
Tecentriq (atezolizumab)	Locally advanced or metastatic urothelial carcinoma that progressed during or following platinum-containing chemotherapy or within 12 months of neoadjuvant or adjuvant treatment with platinum-containing chemotherapy	5/18/2016	4/13/2021
Farydak (panobinostat)	In combination with bortezomib (BTZ) and dexamethasone (DEX) for the treatment of patients with multiple myeloma (MM) who have received at least 2 prior regimens, including BTZ and an immunomodulatory agent	2/23/2015	3/24/2022
Zydelig (idelalisib)	For the treatment of relapsed follicular B-cell Non-Hodgkin Lymphoma (FL) in patients who have received at least 2 prior systemic therapies and relapsed small lymphocytic lymphoma (SLL) in patients who have received at least 2 prior systemic therapies	7/23/2014	2/18/2022
Imbruvica (ibrutinib)	Adult patients with mantle cell lymphoma (MCL) who have received at least one prior therapy	11/13/2013	5/18/2023
Marqibo (vincristine sulfate liposomal)	Adults with Philadelphia (PH) chromosome negative (-) ALL in second relapse or greater relapsed or whose disease has progressed following 2 or greater treatment lines of anti-leukemia therapies	8/9/2012	5/2/2022
Istodax (romidepsin)	Peripheral T-cell lymphoma in patients who have received at least one prior therapy	6/16/2011	7/30/2021
Oforta (fludarabine phosphate)	For adults with B-cell CLL that has not responded to or progressed during or after treatment with at least one standard alkylating agent containing regimen	12/18/2008	12/31/2011
Avastin (bevacizumab)	In combination with paclitaxel for patients who have not received chemotherapy for metastatic HER2 negative breast cancer	2/22/2008	11/18/2011
Bexxar (tositumomab and iodine i 131 tositumomab)	For patients with relapsed or refractory low-grade follicular or transformed CD20+ NHL who have not received rituximab	12/22/2004	10/23/2013
Iressa (gefitinib)	As monotherapy for locally advanced or metastatic NSCLC after failure of platinum-based and docetaxel chemotherapy	5/5/2003	4/25/2012
Mylotarg (gemtuzumab ozogamicin)	For patients with CD33+ AML in first relapse 60 years of age or older and not candidates for cytotoxic chemotherapy	5/17/2000	11/28/2011
Celebrex (celecoxib)	To reduce the number of adenomatous colorectal polyps in familial adenomatous polyposis patients, as an adjunct to usual care	12/23/1999	6/8/2012

The disconnect between scientific insight and regulatory communication has broader consequences beyond just academic understanding. It directly influences how funding organizations, investors, and policy stakeholders prioritize research directions. When resistance-driven treatment failures are misrepresented as vague “inefficacy,” there is an urgent need to invest in mechanism-based drug design, adaptive trial frameworks, and next-generation diagnostic tools. Most funding agencies and pharmaceutical decision-makers operate on milestone-based metrics, favoring drugs that appear effective at short-term endpoints rather than those confronting long-term resistance, a gap that persists partly because resistance is rarely named in regulatory language. This misalignment skews investment away from critical resistance research, limits innovation in biomarker development, and creates the illusion that failure stems from poor design rather than tumor evolution. Without a coordinated effort to bridge this communication gap, the cancer research field risks repeating avoidable mistakes, wasting resources, and stalling truly transformative discoveries. A stronger integration of molecular resistance data into regulatory summaries, transparent public databases documenting emerging resistance mechanisms, and training for grant reviewers and investors in cancer evolutionary biology could significantly change this trajectory. Failing to act now will not just undermine current pipelines; it will erode future innovation, delay lifesaving treatments, and leave the most scientifically challenging cancers, especially in pediatric and refractory settings, further behind. If we continue treating resistance as a silent factor in failure, we will continue designing therapies for yesterday’s tumors that are lost to the future.

## Data Availability

No datasets were generated or analysed during the current study.

## Abbreviations

FDA: Food and Drug Administration
NCT: National Clinical Trial (identifier at ClinicalTrials.gov)
ABC: ATP-Binding Cassette
ABCB1 (MDR1, P-gp): ATP-Binding Cassette Subfamily B Member 1/Multidrug Resistance Protein 1/P-glycoprotein
AKT: Protein Kinase B
BCL2: B-cell lymphoma 2 (anti-apoptotic protein family)
BCRP (ABCG2): Breast Cancer Resistance Protein
CXCL12 (SDF1): Stromal-Derived Factor 1
ECM: Extracellular Matrix
EGF: Epidermal Growth Factor
EMT: Epithelial-to-Mesenchymal Transition
ERCC1/XRCC1: Excision Repair Cross-Complementation Group 1/X-ray Repair Cross-Complementation Protein 1
ERK: Extracellular signal-regulated kinase
FAK: Focal Adhesion Kinase
HGF: Hepatocyte Growth Factor
HIF-1α: Hypoxia-Inducible Factor 1-alpha
IAP: Inhibitors of apoptosis proteins
IL: Interleukin
JAK/STAT: Janus Kinase/Signal Transducer and Activator of Transcription
MCL1: Myeloid Cell Leukemia 1 (anti-apoptotic protein)
MDR: Multidrug Resistance
MDSC: Myeloid-derived suppressor cell
MGMT-O6: Methylguanine-DNA Methyltransferase
MMP: Matrix metalloproteinase
miRNA (miR): MicroRNA
lncRNA: Long Noncoding RNA
NF-κB: Nuclear Factor kappa-light-chain-enhancer of Activated B Cells
NRF2: Nuclear Factor Erythroid 2–Related Factor 2
PARP1: Poly (ADP-ribose) Polymerase 1
PD-L1: Programmed Death-Ligand 1
PTEN: Phosphatase and Tensin Homolog
RISC: RNA-Induced Silencing Complex
ROS: Reactive Oxygen Species
siRNA: Small interfering RNA
Snail/Slug/ZEB1/2/TWIST: EMT-Associated Transcription Factors
SOX2/Oct4/Nanog: Stemness-Associated Transcription Factors
STAT3: Signal Transducer and Activator of Transcription 3
TET: Ten-eleven translocation (DNA demethylase enzymes)
TGF-β: Transforming Growth Factor Beta
VEGF: Vascular Endothelial Growth Factor
Wnt/β-catenin: Wingless/Integrated Signaling Pathway
YAP/TAZ: Yes-Associated Protein/Transcriptional Coactivator with PDZ-binding Motifs
CAF: Cancer-Associated Fibroblasts
CAMDR: Cell adhesion-mediated drug resistance
CAR-T: Chimeric Antigen Receptor T cells
CSC: Cancer Stem Cell
TAM: Tumor-Associated Macrophages
TME: Tumor Microenvironment
Treg: Regulatory T Cells
ASO: Antisense Oligonucleotide
AuNP: Gold Nanoparticle
CRISPR: Clustered Regularly Interspaced Short Palindromic Repeats
dCas9: Catalytically Dead Cas9
EPR: Enhanced Permeability and Retention
EV: Extracellular Vesicle
HA: Hyaluronic Acid
LNP: Lipid Nanoparticle
MSN: Mesoporous silica nanoparticles
NP: Nanoparticle
PAMAM: Poly(amidoamine) Dendrimers
PEG: Polyethylene Glycol
PLGA: Poly(lactic-co-glycolic acid)
PTT: Photothermal Therapy
SNA: Spherical Nucleic Acid
SPION: Superparamagnetic iron oxide nanoparticles
